# Total synthesis of elansolids B1 and B2

**DOI:** 10.3762/bjoc.13.124

**Published:** 2017-06-28

**Authors:** Liang-Liang Wang, Andreas Kirschning

**Affiliations:** 1 Institute of Organic Chemistry and Center of Biomolecular Drug Research (BMWZ), Leibniz University Hannover, Schneiderberg 1b, 30167 Hannover, Germany

**Keywords:** antibiotics, polyenes, polyketides, Stille reaction, Suzuki reaction, total synthesis

## Abstract

The elansolids A1–A3, B1, and B2 are secondary metabolites formed by the gliding bacterium *Chitinophaga sancti*. They show antibacterial activity against Gram-positive bacteria. A second generation total synthesis of the antibiotic elansolid B1 (**2**) and the first synthesis of elansolid B2 (**3**) are reported. In contrast to previous work, the (*Z,E,Z)*-triene at C10–C15 was assembled by using an optimized C–C cross-coupling sequence with a Suzuki cross-coupling reaction as key step.

## Introduction

**Figure 1:**
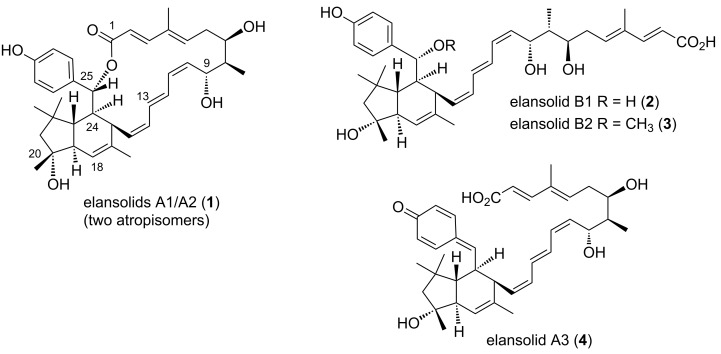
Elansolids A1/A2, B1, B2 and A3 (**1**–**4**).

The elansolids are metabolites from the gliding bacterium *Chitinophaga sancti* (formerly *Flexibacter spec.*) (Figure 1) [1–2]. Elansolid A2 (1*), an atropisomer of elansolid A1 (**1**), showed antibiotic activity against Gram-positive bacteria in the range of 0.2 to 64 µg/mL and cytotoxicity against L929 mouse fibroblast cells with an IC_50_ value of 12 µg/mL. Besides these two macrocylic members also elansolids B1 (**2**) and B2 (**3**) along with A3 (**4**) bearing the unusual *p*-quinone methide unit were isolated from the fermentation broth. All elansolids belong to the group of *trans*-polyketides type I [3–6].

**Scheme 1:**
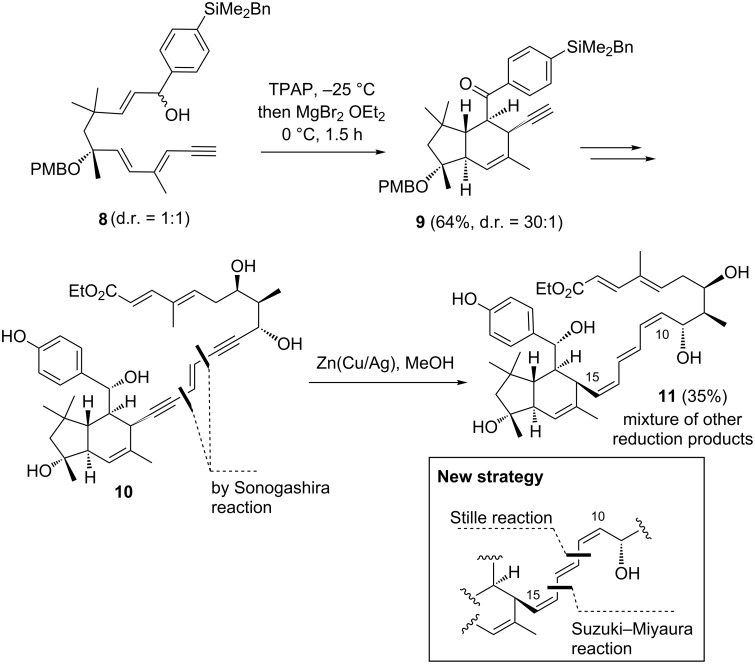
IMDA to generate the tetrahydroindane unit of the elansolids by oxidation of benzyl ether **8** as precursor and construction of the (*Z,E,Z*)-triene unit at C10–C15 (TPAP = tetra-*n*-propylammonium perruthenate(VII); PMB = *p*-methoxybenzyl; Bn = benzyl).

For the first generation total synthesis of elansolid B1 (**2**) we utilized an *endo*-selective intramolecular Diels–Alder (IMDA) cycloaddition as key step to construct the tetrahydroindane unit (Scheme 1) [7]. An enone, derived from allylic alcohol **8** served as precursor to yield tetrahydroindane **9** with excellent diastereocontrol at −25 °C. The major drawback of our first total synthesis of elansolid B1 (**2**) was the installation of the side chain at C1–C13. The synthesis relied on two consecutive Sonogashira–Hagihara cross-coupling reactions that provided the ene–diyne system (C10–C15) **10** in good yield. However, partial hydrogenation (only the zinc–copper couple worked) furnished the desired (*Z,E,Z*)-triene **11** in only low yield (35%) and overreduction was difficult to control. Practically, the reduction was stopped when still substantial amounts of monoreduced product (the alkyne at C10–C11 is reduced preferentially) were present. Consequently, the hydrogenation yielded a mixture of products, which in any case made the separation and isolation a very challenging task.

As continuation of our synthetic investigations on the elansolids, we report an improved second generation approach for generating the carbon chain C1–C13 [8] and for preparing elansolid B1 (**2**). Furthermore, we also describe the first synthesis of elansolid B2 (**3**). The key for improvement was to abandon the two Sonogashira reactions along with the *syn*-reductions of the two alkynes. Instead, we planned to utilize the Suzuki–Miyaura and the Stille reactions and two *Z*-configured vinyl iodides to assemble the (*Z,E,Z*)-triene unit.

## Results and Discussion

**Scheme 2:**
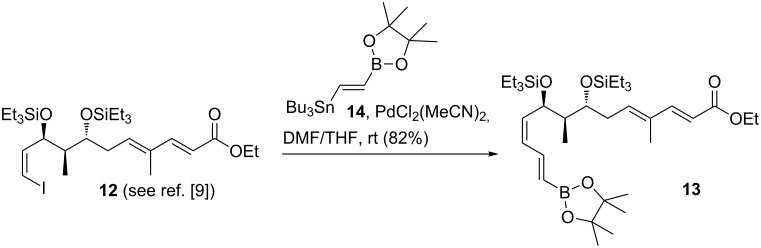
Stille cross-coupling reaction and formation of eastern fragment **13**.

The improved synthesis utilizes the Suzuki–Miyaura cross-coupling reaction to merge the western fragment derived from ketone **9** with the newly designed eastern building block **13**. This fragment was obtained in very good yield from vinyl iodide **12** [9] by a Stille protocol using doubly functionalized alkene **14** which is suited for a sequential cross-coupling strategy (Scheme 2). Under the catalytic conditions, we did not encounter isomerization of the alkene and diene configurations in vinyl boronate **13**.

**Scheme 3:**
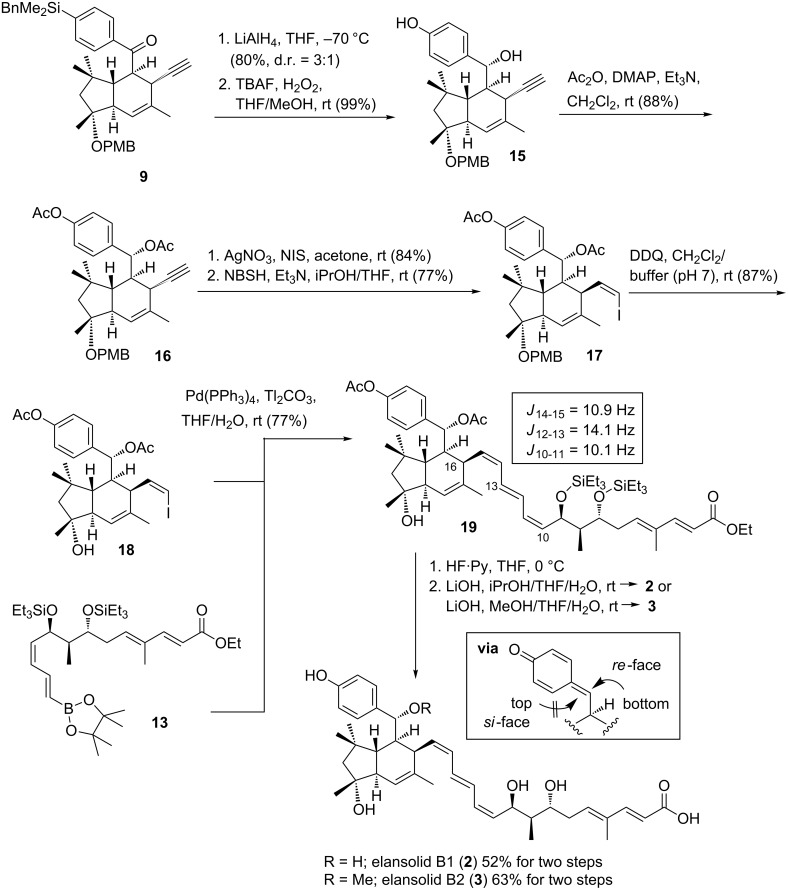
Total synthesis of elansolids B1 (**2**) and B2 (**3**).

The preparation of the newly modified western fragment started from known IMDA product **9** [7], which was first reduced at C-25 (Scheme 3). The two diastereoisomers could be separated by chromatography and the stereochemical assignment of the major isomer was based on X-ray crystallographic analysis [7]. Next, Tamao–Fleming oxidation [10] yielded phenol **15**. The alkyne was transformed into vinyl iodide **17** after *O*-acylation, iodination of the terminal alkyne and finally diimide-mediated *syn*-reduction [11].

Next, DDQ-mediated removal of the PMB protecting group yielded vinyl iodide **18**. The synthesis of both fragments **13** and **18** set the stage for the Suzuki–Miyaura coupling which delivered the desired (*Z*,*E*,*Z*)-configured triene **19**. Again, we did not encounter formation of stereoisomers in the triene unit. The configuration of the triene was unequivocally assigned by analysis of coupling constants (*J*) and by measuring nuclear Overhauser effects (nOe). Finally, desilylation and global saponification of all ester groups in the presence of isopropanol successfully yielded elansolid B1 (**2**). When isopropanol was exchanged by methanol, elansolid B2 (**3**) was generated. Its formation can be rationalized by formation of the intermediate *p*-methide quinone which selectively trapped methanol, exclusively yielding the *R*-isomer at C25. This excellent facial selectivity has been demonstrated, e.g., for anilines as nucleophiles before. It is due to the preferred conformation around the bond at C24–C25 which leads to the efficient shielding of the *si*-face by the two germinal methyl groups at C22 [4–5]. The NMR data determined for both synthetic products were identical with those of authentic samples of elansolid B1 (**2**) and elansolid B2 (**3**) (copies of spectra, see Supporting Information File 1).

## Conclusion

In conclusion, we describe an improved second generation synthesis of the highly active antibiotic elansolid B1 (**2**). The improvements are mainly associated with the preparation of the triene unit at C10–C15 by utilizing the Stille and the Suzuki–Miyaura cross-coupling reactions as well as the highly versatile difunctionalized building block **14**. In principal, the synthesis sheds light on how such (*Z,E,Z*)-configured triene units are ideally be constructed, clearly demonstrating that enediynes are less preferred precursors for such structural elements. It has to be noted that there is precedence in the literature for the use of the Suzuki–Miyaura cross-coupling reaction as key step to assemble differently configured trienes present in polyketides [12–15].

Furthermore, we show how the intermediate *p*-methide quinone can be exploited to also prepare elansolid B2 (**3**). The improved synthesis allows more easily preparing analogues of the elansolids for further biological evaluation.

## Experimental

### General information:

^1^H NMR spectra were recorded at 400 MHz or 500 MHz, respectively, and ^13^C NMR spectra were recorded at 100 MHz or 125 MHz, respectively, with a Bruker Avance 400, DPX 400 or DRX 500. Chemical shift values of NMR data are reported as values in ppm relative to the (residual undeuterated) solvent signal as internal standard. Multiplicities for ^1^H NMR signals are described using the following abbreviations: s = singlet, d = doublet, t = triplet, q = quartet, m = multiplet; where appropriate with the addition of b = broad. Mass spectra were obtained with a type LCT (ESI) (Micromass) equipped with a lockspray dual ion source in combination with a Waters Alliance 2695 LC system, or with a type QTOF premier (Micromass) spectrometer (ESI mode) in combination with a Waters Acquity UPLC system equipped with a Waters BEH C18 1.7 μm (SN 01473711315545) column (solvent A: water + 0.1% (v/v) formic acid, solvent B: MeOH + 0.1% (v/v) formic acid; flow rate = 0.4 mL/min; gradient (*t* [min]/solvent B [%]): (0:5) (2.5:95) (6.5:95) (6.6:5) (8:5)). Ion mass signals (*m*/*z*) are reported as values in atomic mass units. Optical rotations were measured on a Perkin-Elmer polarimeter type 341 or 241 in a quartz glass cuvette at *l* = 589 nm (Na D-line). The optical rotation is given in [° mL·g^−1^·dm^−1^] with *c* = 1 corresponding to 10 mg mL^−1^. Preparative HPLC was operated at a Merck Hitachi LaChrome HPLC (Pump L7150 or L7100, Interface D-7000, Diode Array Detector L-7450), respectively, at a Beckmann system Gold HPLC (Solvent Module 125, Detector 166). Solvents, columns, operating procedures and retention times are given with the corresponding experimental and analytical data.

All reactions were performed under an argon atmosphere unless otherwise stated. Glassware was dried by heating under vacuum followed by flushing with argon gas prior to use. Dry solvents were obtained after filtration through drying columns on a M. Braun solvent purification system or purchased form commercial providers. The synthesis of building blocks **9** [7] and **12** [9] was reported before.

### Synthetic experiments

#### Synthesis of boronate **13**

A flame dried round bottom flask equipped with a stirring bar was charged with vinyl iodide **12** (10 mg, 16 µmol, 1.0 equiv) and boronate **14** (10.68 mg, 24 µmol, 1.5 equiv) in DMF (0.3 mL) and THF (0.1 mL). To this stirred solution PdCl_2_(MeCN)_2_ (1.25 mg, 4.8 µmol, 0.3 equiv) was added at room temperature and stirring was continued for 8 h. The volatiles were removed in vacuo and the residue was purified by silica gel chromatography to afford vinyl boronate **13** (8.5 mg, 12.3 µmol, 82%). *R*_f_ = 0.40 (PE/EtOAc 10:1, visualized using an anisaldehyde stain or UV), [α]_D_^20^ = −34.5 (*c* 0.8, CH_2_Cl_2_); ^1^H NMR (400 MHz, CDCl_3_, CHCl_3_ = 7.26 ppm) δ 7.32 (d, *J =* 15.6 Hz, 1H), 7.25 (d, *J =* 11.4, 17.5 Hz, 1H), 6.09 (t, *J =* 11.1 Hz, 1H), 5.97 (dd, *J =* 7.1 Hz, 1H), 5.78 (d, *J =* 15.5 Hz, 1H), 5.61 (d, *J =* 17.4 Hz, 1H), 5.57 (t, *J =* 10.2 Hz, 1H), 4.63 (dd, *J =* 6.4, 9.2 Hz, 1H), 4.22 (q, *J =* 7.1 Hz, 2H), 3.84–3.80 (m, 1H), 2.42–2.34 (m, 1H), 2.27–2.23 (m, 1H), 1.76 (s, 3H), 1.74–1.71 (m, 1H), 1.33 (t, *J =* 7.2 Hz, 3H), 1.28 (s, 12H), 0.97–0.93 (m, 21H), 0.59 (q, *J =* 8.0Hz, 12H); ^13^C NMR (100 MHz, CDCl_3_ = 77.16 ppm) δ 167.6, 149.6, 143.8, 139.6, 137.4, 133.6, 130.6, 123.3, 115.4, 83.2, 72.5, 69.8, 60.1, 47.1, 32.6, 24.8, 24.7, 14.3, 12.6, 9.7, 7.0, 6.9, 5.2, 5.1; HRMS (ESI) *m/z*: [M + Na]^+^ calculated for C_35_H_65_BO_6_Si_2_Na, 671.4310; found, 671.4312.

#### Reduction of ketone **9** and formation of benzyl alcohol

A solution of ketone **9** (466.2 mg, 0.79 mmol, 1.0 equiv) in THF (3 mL) was added to a suspension of LiAlH_4_ (599.2 mg, 15.79 mmol, 20 equiv) in THF (12 mL) at −70 °C. After stirring for 3 d at this temperature the reaction was terminated by slow addition of a saturated potassium sodium tartrate solution. The reaction mixture was vigorously stirred for 1 h at room temperature. The layers were seperated and the aqueous phase was extracted with ethyl acetate. The combined organic extracts were dried over Na_2_SO_4_, filtered and concentrated under reduced pressure. The resulting benzyl alcohol (371.7 mg, 0.63 mmol, 80%, d.r. = 3:1) was obtained after flash column chromatography. Separation of diastereomers was achieved by preparative HPLC (C18 ISIS-SP) (gradient H_2_O/MeOH 30:70 to 0:100 {0–80 min}, 15 mL/min, *t*_R_ = 67.6 min). *R*_f_ = 0.6 (PE/EtOAc 5:1, visualized using anisaldehyde stain or UV), [α]_D_^20^ = +28.9 (*c* 0.82, CH_2_Cl_2_); ^1^H NMR (400 MHz, CDCl_3_, CHCl_3_ = 7.26 ppm) δ 7.48 (d, *J =* 8.0 Hz, 2H), 7.42 (d, *J =* 7.9 Hz, 2H), 7.30 (d, *J =* 9.8 Hz, 2H), 7.19 (t, *J =* 7.5 Hz, 2H), 7.08 (t, *J =* 7.3 Hz, 1H), 6.94–6.89 (m, *J =* 6.8 Hz, 4H), 5.60 (s, 1H), 5.29 (dd, *J =* 2.9, 11.0 Hz, 1H), 4.47 (d, *J =* 10.8 Hz, 1H), 4.40 (d, *J =* 10.8 Hz, 1H), 4.27 (d, *J =* 11.1 Hz, 1H, OH), 3.83 (s, 3H), 2.87 (d, *J =* 10.7 Hz, 1H), 2.57 (br, 1H), 2.48 (d, *J =* 2.4 Hz, 1H), 2.33 (s, 2H), 2.20 (ddd, *J =* 3.5, 4.6, 11.4 Hz, 1H), 2.10 (d, *J =* 14.0 Hz, 1H), 2.02 (dd, *J =* 11.8, 12.0 Hz, 1H), 1.69 (s, 3H), 1.69 (d, *J =* 13.6 Hz, 1H), 1.40 (s, 3H), 1.32 (s, 3H), 1.29 (s, 3H), 0.29 (s, 6H); ^13^C NMR (100 MHz, CDCl_3_ = 77.16 ppm) δ 158.9, 144.3, 139.7, 136.6, 133.8, 133.2, 131.8, 128.7, 128.3, 128.1, 124.7, 124.1, 123.7, 113.8, 86.7, 81.3, 73.9, 73.5, 64.9, 55.8, 55.3, 52.9, 46.6, 45.3, 38.0, 33.4, 31.1, 26.3, 24.2, 22.7, 21.3, −3.4, −3.3; HRMS (ESI) *m/z*: [M + Na]^+^ calculated for C_39_H_48_O_3_SiNa, 615.3270; found, 615.3270.

#### Synthesis of phenol **15**

To a solution of the benzyl alcohol described above (124.0 mg, 0.21 mmol, 1.0 equiv) in THF (0.75 mL) was added a solution of TBAF (*c* = 1.0 M in THF, 0.84 mL, 4.0 equiv) and the mixture was stirred for 15 min. Methanol (2.23 mL), KHCO_3_ (41.85 mg, 0.42 mmol, 2.0 equiv) and H_2_O_2_ (35% in H_2_O, 0.36 mL, 4.18 mmol, 20 equiv) were sequentially added and stirring was continued overnight. Then, the reaction was terminated by slow addition of a saturated, aqueous Na_2_S_2_O_3_ solution and the aqueous layer was extracted with ethyl acetate. The combined organic extracts were dried over Na_2_SO_4_ filtered and concentrated in vacuo. Purification by flash column chromatography gave phenol **15** (96.0 mg, 0.20 mol, 99%) as a colorless solid. *R*_f_ = 0.20 (PE/EtOAc 3:1, visualized using an anisaldehyde stain or UV), [α]_D_^20^ = +26.1 (*c* 0.7, CH_2_Cl_2_); ^1^H NMR (400 MHz, CDCl_3_, CHCl_3_ = 7.26 ppm) δ 7.31–7.28 (m, 4H), 6.90 (d, *J =* 8.7 Hz, 2H), 6.86 (d, *J =* 8.6 Hz, 2H), 5.59 (s, 1H), 5.25 (dd, *J =* 3.0, 10.9 Hz, 1H), 4.97 (s, 1H, OH), 4.47 (d, *J =* 10.8 Hz, 1H), 4.39 (d, *J =* 10.8 Hz, 1H), 4.22 (d, *J =* 10.9 Hz, 1H), 3.82 (s, 3H), 2.85 (dd, *J =* 1.8, 12.4 Hz, 1H), 2.60 (br, 1H), 2.45 (d, *J =* 2.5 Hz, 1H), 2.13 (ddd, *J =* 3.8, 4.4, 11.1Hz, 1H), 2.08 (d, *J =* 14.0 Hz, 1H), 2.00 (t, *J =* 11.8 Hz, 1H), 1.68 (s, 3H), 1.67 (d, *J =* 13.7 Hz, 1H), 1.38 (s, 3H), 1.30 (s, 3H), 1.28 (s, 3H); ^13^C NMR (100 MHz, CDCl_3_ = 77.16 ppm) δ 158.9, 154.4, 135.5, 133.2, 131.8, 128.7, 126.6, 123.7, 115.2, 113.8, 86.8, 81.3, 73.9, 73.1, 64.9, 55.7, 55.3, 52.9, 46.7, 45.4, 38.0, 33.3, 31.1, 24.2, 22.7, 21.3; HRMS (ESI) *m/z*: [M + H]^+^ calculated for C_30_H_37_O_4_, 461.2692; found, 461.2693.

#### Synthesis of acetyl ester **16**

A round bottom flask equipped with a magnetic stirring bar was sequentially charged with alcohol **15** (89.6 mg, 0.195 mmol, 1.0 equiv), CH_2_Cl_2_ (2 mL), DMAP (23.82 mg, 0.196 mmol, 1.0 equiv), Et_3_N (0.204 mL, 1.46 mmol, 7.4 equiv) and Ac_2_O (0.092 mL, 0.973 mmol, 4.0 equiv). After stirring at ambient temperature for 2 d the reaction was terminated by addition of H_2_O and extracted with Et_2_O several times. The combined organic phases were dried over anhydrous Na_2_SO_4_, filtered, concentrated in vacuo. The residue was purified by silica gel column chromatography to give compound **16** (93 mg, 0.17 mmol, 88%). *R*_f_ = 0.5 (PE/EtOAc 3:1, visualized using an anisaldehyde or UV), [α]_D_^20^ = +35.8 (*c* 0.5, CH_2_Cl_2_); ^1^H NMR (400 MHz, CDCl_3_, CHCl_3_ = 7.26 ppm) δ 7.41 (d, *J =* 8.6 Hz, 2H), 7.27 (d, *J =* 9.0 Hz, 2H), 7.11 (d, *J =* 8.6 Hz, 2H), 6.89 (d, *J =* 8.7 Hz, 2H), 6.01 (d, *J =* 3.2 Hz, 1H), 5.54 (s, 1H), 4.44 (d, *J =* 10.8 Hz, 1H), 4.37 (d, *J =* 10.8 Hz, 1H), 3.82 (s, 3H), 2.84 (dd, *J =* 1.8, 12.3 Hz, 1H), 2.65 (br, 1H), 2.32 (s, 3H), 2.24 (d, *J =* 2.6 Hz, 1H), 2.14 (s, 3H), 2.10–2.06 (m, 1H), 2.03–2.00 (m, 2H), 1.73 (s, 3H), 1.60 (d, *J =* 14.0 Hz, 1H), 1.28 (s, 3H), 1.24 (s, 3H), 1.11 (s, 3H); ^13^C NMR (100 MHz, CDCl_3_ = 77.16 ppm) δ 170.3, 169.3, 158.9, 149.9, 137.0, 134.5, 131.7, 128.7, 126.9, 122.7, 121.5, 113.8, 85.4, 81.0, 74.9, 71.0, 64.9, 55.7, 55.3, 53.5, 46.1, 45.8, 37.9, 34.6, 29.9, 23.8, 22.8, 21.6, 21.2, 21.1; HRMS (ESI) *m/z*: [M + Na]^+^ calculated for C_34_H_40_O_6_Na, 567.2723; found, 567.2728.

#### Synthesis of vinyl iodide **17**

To a stirred solution of compound of **16** (88 mg, 0.162 mmol, 1.0 equiv) in acetone (4 mL) was added AgNO_3_ (8.3 mg, 0.05 mmol, 0.3 equiv) and *N*-iodosuccinimide (40.0 mg, 0.18 mmol, 1.1 equiv) at ambient temperature. The resulting mixture was stirred for 1.5 h and concentrated in vacuo. The residue was purified by silica gel column chromatography to give the corresponding alkynyl iodide (91.0 mg, 0.136 mmol, 84%). This product (81.9 mg, 0.12 mmol, 1.0 equiv) was dissolved in a mixture of THF (1.1 mL) and iPrOH (1.1 mL). Et_3_N (0.026 mL, 0.183 mmol, 1.5 equiv) and 2-nitrobenzenesulfonyl hydrazide (34.5 mg, 0.159 mmol, 1.3 equiv) were sequentially added. After stirring for 20 h, Et_3_N (0.026 mL, 0.183 mmol, 1.5 equiv) and 2-nitrobenzenesulfonyl hydrazide (17.25 mg, 0.079 mmol, 0.7 equiv) were added and the reaction mixture was stirred for additional 5 h. The volatiles were then removed in vacuo at ambient temperature. The residue was purified by silica gel column chromatography to give vinyl iodide **17** (62.0 mg, 0.092 mmol, 77%). *R*_f_ = 0.60 (PE/EtOAc 3:1, visualized using an anisaldehyde stain or UV), [α]_D_^20^ = +14.1 (*c* 0.95, CH_2_Cl_2_); ^1^H NMR (400 MHz, CDCl_3_, CHCl_3_ = 7.26 ppm) δ 7.28 (d, *J =* 8.6 Hz, 2H), 7.17 (d, *J =* 8.6 Hz, 2H), 7.11 (d, *J =* 8.6 Hz, 2H), 6.90 (d, *J =* 8.6 Hz, 2H), 6.43 (dd, *J =* 7.8, 9.4 Hz, 1H), 6.29 (d, *J =* 7.5 Hz, 1H), 6.03 (d, *J =* 2.5 Hz, 1H), 5.56 (s, 1H), 4.45 (d, *J =* 10.8 Hz, 1H), 4.38 (d, *J =* 10.8 Hz, 1H), 3.82 (s, 3H), 2.89–2.83 (m, 2H), 2.37 (ddd, *J =* 2.9, 3.7, 10.9 Hz, 1H), 2.31 (s, 3H), 2.15 (s, 3H), 2.07 (d, *J =* 14.0 Hz, 1H), 1.79 (t, *J =* 11.8 Hz, 1H), 1.64 (s, 3H), 1.59 (d, *J =* 14.0 Hz, 1H), 1.30 (s, 3H), 1.24 (s, 3H), 1.09 (s, 3H); ^13^C NMR (100 MHz, CDCl_3_ = 77.16 ppm) δ 169.9, 169.3, 158.9, 149.6, 141.9, 136.1, 135.2, 131.7, 128.7, 126.7, 123.7, 121.9, 113.8, 80.9, 80.1, 75.5, 64.9, 55.9, 55.3, 53.4, 46.6, 46.1, 46.0, 38.3, 30.1, 23.5, 22.7, 22.2, 21.3, 21.2; HRMS (ESI) *m/z*: [M + Na]^+^ calculated for C_34_H_41_O_6_INa, 695.1846; found, 695.1837.

#### Synthesis of vinyl iodide **18**

DDQ (56.5 mg, 0.25 mmol, 3.0 equiv) was added to a stirred solution of **17** (55.8 mg, 0.083 mmol, 1.0 equiv) in CH_2_Cl_2_ (4.5 mL)/pH 7.0 phosphate buffer (0.45 mL) at 0 °C. After stirring for 1.5 h, the reaction mixture was terminated by addition of a saturated, aqueous NaHCO_3_ solution. The aqueous solution was extracted with Et_2_O. The combined, organic phases were dried over anhydrous Na_2_SO_4_, filtered, concentrated in vacuo and the crude product was purified by silica gel column chromatography to furnish the title vinyl iodide **18** (40.0 mg, 0.072 mol, 87%). *R*_f_ = 0.25 (PE/EtOAc 3:1, visualized using an anisaldehyde or UV), [α]_D_^20^ = +36.7 (*c* 0.86, CH_2_Cl_2_); ^1^H NMR (400 MHz, CDCl_3_, CHCl_3_ = 7.26 ppm) δ 7.17 (d, *J =* 8.6 Hz, 2H), 7.11 (d, *J =* 8.6 Hz, 2H), 6.40 (dd, *J =* 7.6, 9.6 Hz, 1H), 6.28 (d, *J =* 7.6 Hz, 1H), 6.03 (d, *J =* 2.3 Hz, 1H), 5.51 (s, 1H), 2.84 (dd, *J =* 4.1, 9.6 Hz, 1H), 2.70 (d, *J =* 12.7 Hz, 1H), 2.34 (ddd, *J =* 2.8, 4.0, 11.3 Hz, 1H), 2.31 (s, 3H), 2.15 (s, 3H), 1.84 (d, *J =* 14.2 Hz, 1H), 1.77 (d, *J =* 14.2 Hz, 1H), 1.74 (t, *J =* 11.9 Hz, 1H), 1.65 (s, 3H), 1.30 (s, 3H), 1.19 (s, 3H), 1.07 (s, 3H); ^13^C NMR (100 MHz, CDCl_3_ = 77.16 ppm) δ 169.9, 169.3, 149.6, 141.8, 136.1, 135.7, 126.7, 122.4, 121.9, 80.1, 76.2, 75.4, 60.1, 54.8, 46.4, 46.3, 45.9, 38.1, 30.2, 26.4, 23.9, 22.2, 21.2, 21.1; HRMS (ESI) *m/z*: [M + Na]^+^ calculated for C_26_H_33_O_5_INa, 575.1270; found, 575.1272.

#### Synthesis of triene **19**

To a stirred solution of vinyl iodide **18** (21 mg, 40.2 µmol, 1.0 equiv) and boronate **13** (39.12 mg, 60.0 µmol, 1.5 equiv) in THF (5 mL) and H_2_O (1.25 mL) were sequentially added thallium(I) carbonate (33.92 mg, 72.0 µmol, 1.8 equiv) and Pd(PPh_3_)_4_ (13.93 mg, 12.0 µmol, 0.3 equiv) at room temperature. The reaction mixture was stirred for 4 h and then H_2_O was added. The aqueous solution was extracted with Et_2_O. The combined, organic phases were dried over anhydrous Na_2_SO_4_, filtered, concentrated in vacuo and the crude product was purified by silica gel column chromatography to furnish the title triene **19** (29.3 mg, 31.0 µmol, 77%). *R*_f_ = 0.4 (PE/EtOAc 2:1, visualized using an anisaldehyde or UV), [α]_D_^20^ = +77.2 (*c* 0.53, CH_2_Cl_2_); ^1^H NMR (400 MHz, acetone-*d*_6_, acetone-*d*_5_ = 2.05 ppm) δ 7.32 (d, *J =* 15.7 Hz, 1H, H3), 7.19 (d, *J =* 8.5 Hz, 2H, H27), 7.00 (d, *J =* 8.5 Hz, 2H, H28), 6.64 (dd, *J =* 11.7, 14.1 Hz, 1H, H12), 6.17 (dd, *J =* 10.9, 11.2 Hz, 1H, H14), 6.11–6.04 (m, 3H, H5, H11, H13), 6.01 (d, *J =* 2.6 Hz, 1H, H25), 5.83 (d, *J =* 15.7 Hz, 1H, H2), 5.78 (d, *J =* 10.4, 10.7 Hz, 1H, H15), 5.55 (s,1H, H18), 5.48 (t, *J =* 10.1 Hz, 1H, H10), 4.71 (dd, *J =* 6.6, 9.2 Hz, 1H, H9), 4.14 (q, *J =* 7.1 Hz, 2H, H1’), 4.05–4.00 (m, 1H, H7), 2.87–2.88 (m, 1H, H16), 2.74 (d, *J =* 12.6 Hz, 1H, H19), 2.49 (t, *J =* 6.9 Hz, 2H, H6), 2.39 (ddd, *J =* 3.3, 3.5, 11.4 Hz, 1H, H24), 2.27 (s, 3H, H12’), 2.12 (s, 3H, H10’), 1.93 (dd, *J =* 11.8, 11.9 Hz, 1H, H23), 1.89 (s, 3H, H3’), 1.84 (d, *J =* 13.6 Hz, 1H, H21b), 1.83–1.80 (m, 1H, H8), 1.73 (d, *J =* 13.9 Hz, 1H, H21a), 1.45 (s, 3H, H5’), 1.30 (s, 3H, H6’), 1.23 (t, *J =* 7.1 Hz, 3H, H2’), 1.14 (s, 3H, H8’), 1.08 (d, *J =* 6.8 Hz, 3H, H4’), 1.07 (s, 3H, H7’), 1.04–0.99 (m, 18H, H14’, H16’), 0.70–0.58 (m, 12H, H13’, H15’); ^13^C NMR (400 MHz, acetone-*d*_6_ = 29.8 and 206.3 ppm) δ 169.4 (C9’), 168.7 (C11’), 166.4 (C1), 149.8 (C29), 148.8 (C3), 139.3 (C5), 137.1 (C26), 134.9 (C17), 134.5 (C15), 133.8 (C4), 133.2 (C10), 131.6 (C13), 129.4 (C11), 127.1 (C12), 126.5 (C27), 125.6 (C14), 122.7 (C18),121.3 (C28), 115.9 (C2), 75.0 (C25) , 74.8 (C20), 72.9 (C7), 70.1 (C9), 60.2 (C21), 59.6 (C1’), 55.1 (C19), 47.4 (C8), 46.6 (C24), 44.7 (C23), 40.8 (C16), 37.5 (C22), 32.6 (C6), 29.8 (C7’), 25.7 (C8’), 23.6 (C6’), 20.6 (C5’), 20.3 (C12’), 20.1 (C10’), 13.7 (C2’), 11.9 (C3’), 9.5 (C4’), 6.5 (C14’), 6.3 (C16’), 4.9 (C13’,15’); HRMS (ESI) *m/z*: [M + Na]^+^ calculated for C_55_H_86_O_9_Si_2_Na, 969.5708; found, 969.5707.

#### Synthesis of elansolid B1 (**2**)

Polyene **19** (2.65 mg, 2.79 µmol, 1.0 equiv) was dissolved in THF (0.5 mL) and cooled to 0 °C. A solution of hydrogen fluoride pyridine complex (0.5 mL) prepared by mixing hydrogen fluoride pyridine (2 mL; hydrogen fluoride ≈70 %) with pyridine (5.6 mL) in THF (9.8 mL) at 0 °C. The reaction mixture was stirred for 1 h at this temperature and the reaction was terminated by addition of a saturated bicarbonate solution. The aqueous solution was extracted with Et_2_O for three times. The combined organic phases were dried over Na_2_SO_4_, filtered and concentrated under reduced pressure to afford the corresponding diol suitably pure for directly being employed in the next step. An aqueous solution of LiOH (1 M, 0.3 mL, 107 equiv) was added to crude diol in iPrOH (0.3 mL) and THF (0.3 mL) at room temperature. After stirring for 5 h, the reaction was terminated by slowly adding HCl (1 N, 0.24 mL), phosphate buffer (pH 7, 0.1 mL) and MeOH (0.3 mL). The crude mixture was directly subjected to HPLC (C18 ISIS-SP) (MeOH: H_2_O/50 mM NH_4_OAc 70:30 to MeOH:H_2_O/50 mM NH_4_OAc 100:0 {0–70 min}, 3.0 mL/min) to give elansolid B1 (**2**) (0.88 mg, 1.45 µmol, 52% over two steps, *t*_R_ = 47.8 min). [α]_D_^20^ = +176.0 (*c* 0.05, MeOH); ^1^H NMR (500 MHz, acetone-*d*_6_; acetone-*d*_5_ = 2.05 ppm) δ 7.35 (d, *J =* 15.6 Hz, 1H, H3), 7.14 (d, *J =* 8.3 Hz, 2H, H27), 6.75 (d, *J =* 8.5 Hz, 2H, H28), 6.57 (dd, *J =* 12.2, 13.6 Hz, 1H, H12), 6.17 (t, *J =* 7.23 Hz, 1H, H5), 6.09–6.02 (m, 2H, H13, H14), 5.99 (dd, *J =* 10.8, 11.0 Hz, 1H, H11), 5.84 (d, *J =* 15.7 Hz, 1H, H2), 5.70 (*t*, *J =* 10.5 Hz, 1H, H15), 5.55 (dd, *J =* 9.5, 10.9 Hz, 1H, H10), 5.52 (s, 1H, H18), 5.18 (d, *J =* 2.5 Hz, 1H, H25), 4.94 (ddd, *J =* 0.9, 3.6, 8.6 Hz, 1H, H9), 3.87–3.84 (m, 1H, H7), 2.92 (dd, *J =* 4.0, 10.8 Hz, 1H, H16), 2.69 (d, *J =* 2.6 Hz, 1H, H19), 2.59 (ddd, *J =* 4.2, 6.8, 15.3 Hz, 1H, H6a), 2.51–2.47 (m, 1H, H6b), 2.22–2.18 (m, 1H, H24), 1.99–1.95 (m, 1H, H23), 1.88 (s, 3H, H1’), 1.83–1.80 (m, 1H, H8), 1.83 (d, *J =* 13.8 Hz, 1H, H21a), 1.73 (d, *J =* 13.8 Hz, 1H, H21b), 1.44 (dd, *J =* 1.4, 2.2 Hz, 3H, H3’), 1.30 (s, 3H, H5’), 1.29 (s, 3H, H6’), 1.14 (s, 3H, H4’), 1.03 (d, *J =* 7.0 Hz, 3H, H2’); ^13^C NMR (125 MHz, acetone-*d*_6_ = 29.8 and 206.3 ppm) δ 167.5 (C1), 155.7 (C29), 149.4 (C3), 138.9 (C5), 135.2 (C26, C17), 134.8 (C15), 133.8 (C4), 132.9 (C10), 130.9 (C13), 129.3 (C11), 127.5 (C12), 127.0 (C27), 126.5 (C14), 122.5 (C18), 115.9 (C2), 114.5 (C28), 74.9 (C20), 73.0 (C7), 72.2 (C25), 68.5 (C9), 60.5 (C21), 55.1 (C19), 48.4 (C24), 44.5 (C23), 44.0 (C8), 40.4 (C16), 37.7 (C22), 34.1 (C6), 30.9 (C5’), 25.7 (C4’), 23.8 (C6’), 20.6 (C3’), 11.8 (C1’), 10.7 (C2’); HRMS (ESI) *m/z*: [M + Na]^+^ calculated for C_37_H_50_O_7_Na, 629.3454; found, 629.3463.

#### Synthesis of elansolid B2 (**3**)

In aqueous solution of LiOH (1 M, 0.3 mL, 107 equiv) was added to the crude diol (3.1 µmol) described for the synthesis of elansolid B1 (**2**) in MeOH (0.3 mL) and THF (0.3 mL) at room temperature. After stirring for 5 h, the reaction was terminated by slowly adding HCl (1 N, 0.24 mL), phosphate buffer (pH 7, 0.1 mL) and MeOH (0.3 mL). The crude mixture was directly subjected to HPLC (C18 ISIS-SP) (MeOH: H_2_O/50 mM NH_4_OAc 70:30 to MeOH:H_2_O/50 mM NH_4_OAc 100:0 {0–70 min}, 3.0 mL/min) to give elansolid B2 (**3**) (1.2 mg, 1.93 µmol, 63% over two steps, *t*_R_ = 63.4 min). [α]_D_^20^ = +262.1 (*c* 0.087, MeOH); ^1^H NMR (400 MHz, acetone-*d*_6_; acetone-*d*_5_ = 2.05 ppm) δ 7.35 (d, *J =* 15.7 Hz, 1H, H3), 7.07 (d, *J =* 8.4 Hz, 2H, H27), 6.77 (d, *J =* 8.5 Hz, 2H, H28), 6.63 (dd, *J =* 11.5, 14.6 Hz, 1H, H12), 6.34 (dd, *J =* 11.5, 14.6 Hz, 1H, H13), 6.18 (t, *J =* 7.2 Hz, 1H, H5), 6.13 (t, *J =* 11.2 Hz, 1H, H11), 6.02 (t, *J =* 11.2 Hz, 1H, H14), 5.82 (d, *J =* 15.7 Hz, 1H, H2), 5.68 (t, *J =* 10.8 Hz, 1H, H15), 5.57 (dd, *J =* 9.7, 10.1 Hz, 1H, H10), 5.48 (s, 1H, H18), 4.99 (ddd, *J =* 0.9, 3.5, 8.6 Hz, 1H, H9), 4.66 (d, *J =* 2.4 Hz, 1H, H25), 3.88–3.84 (m, 1H, H7), 3.12 (s, 3H, H7’), 2.90 (dd, *J =* 3.3, 10.6 Hz, 1H, H16), 2.64–2.62 (m, 1H, 19H), 2.60–2.58 (m, 1H, H6a), 2.52–2.44 (m, 1H, H6b), 2.01–1.97 (m, 1H, H24), 1.95–1.91 (m, 1H, H23), 1.89 (s, 3H, H1’), 1.85–1.82 (m, 1H, H8), 1.82 (d, *J =* 13.5 Hz, 1H, H21a), 1.72 (d, *J =* 13.8 Hz, 1H, H21b), 1.45 (dd, *J =* 1.3, 2.1 Hz, 3H, H3’), 1.29 (s, 3H, H5’), 1.21 (s, 3H, H6’), 1.12 (s, 3H, H4’), 1.03 (d, *J =* 7.0 Hz, 3H, H2’); ^13^C NMR (100 MHz, acetone-*d*_6_ = 29.8 and 206.3 ppm) δ 167.3 (C1), 156.2 (C29), 149.4 (C3), 139.0 (C5), 135.5 (C15), 135.3 (C17), 133.8 (C4), 132.5 (C10), 131.6 (C13), 131.0 (C26), 129.4 (C11), 128.3 (C27), 126.8 (C12), 124.3 (C14), 122.5 (C18), 115.7 (C2), 114.7 (C28), 82.9 (C25), 74.8 (C20), 73.0 (C7), 68.5 (C9), 60.4 (C21), 55.6 (C7’), 55.2 (C19), 48.8 (C24), 44.8 (C23), 44.0 (C8), 40.3 (C16), 37.6 (C22), 34.2 (C6), 30.4 (C6’), 25.7 (C4’), 23.7 (C5’), 20.9 (C3’), 11.8 (C1’), 10.8 (C2’); HRMS (ESI) *m/z*: [M + Na]^+^ calculated for C_38_H_52_O_7_Na, 643.3611; found, 643.3611.

## Supporting Information

File 1 ^1^H and ^13^C NMR spectra of synthesized compounds.
