# Supplementary material for: Total synthesis of elansolids B1 and B2
Source: Beilstein J Org Chem. 2017 Jun 28;13:1280–7. doi: 10.3762/bjoc.13.124 (PMC5496564; doi:10.3762/bjoc.13.124)
Supplement: File 1 — 1H and 13C NMR spectra of synthesized compounds. [file Beilstein_J_Org_Chem-13-1280-s001.pdf]

Supporting Information

for

Total synthesis of elansolids B1 and B2

Liang-Liang Wang and Andreas Kirschning*

Address: Institute of Organic Chemistry and Center of Biomolecular Drug Research (BMWZ), Leibniz University Hannover, Schneiderberg 1b, 30167 Hannover, Germany

Email: Andreas Kirschning - andreas.kirschning@oci.uni-hannover.de

*Corresponding author

^{1}H and ^{13}C NMR spectra of synthesized compounds

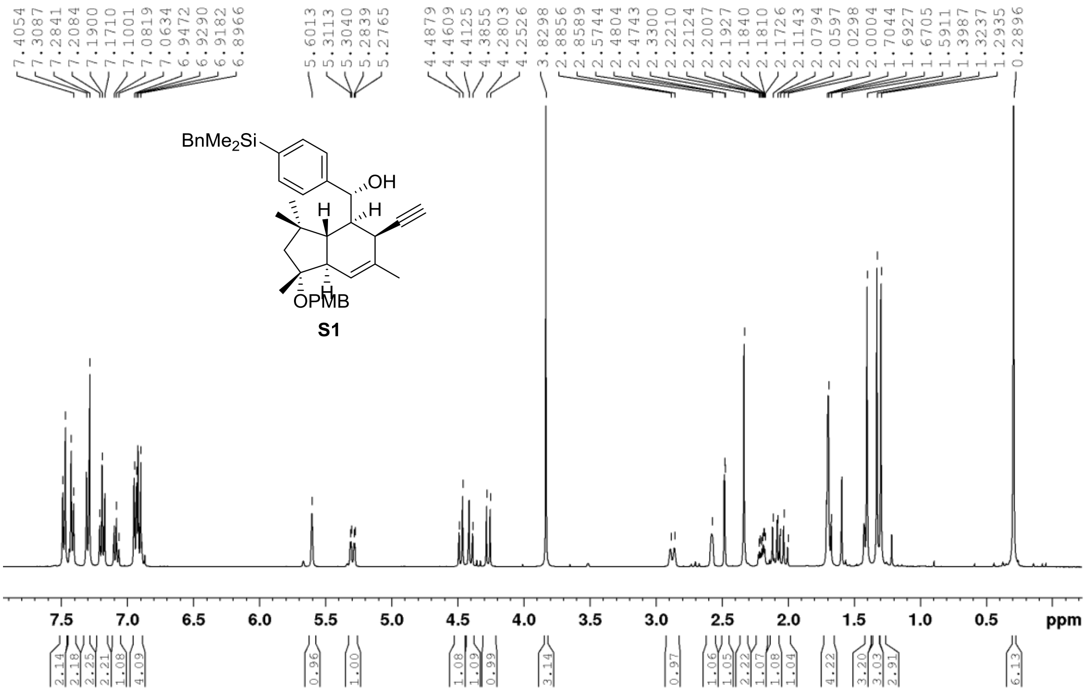

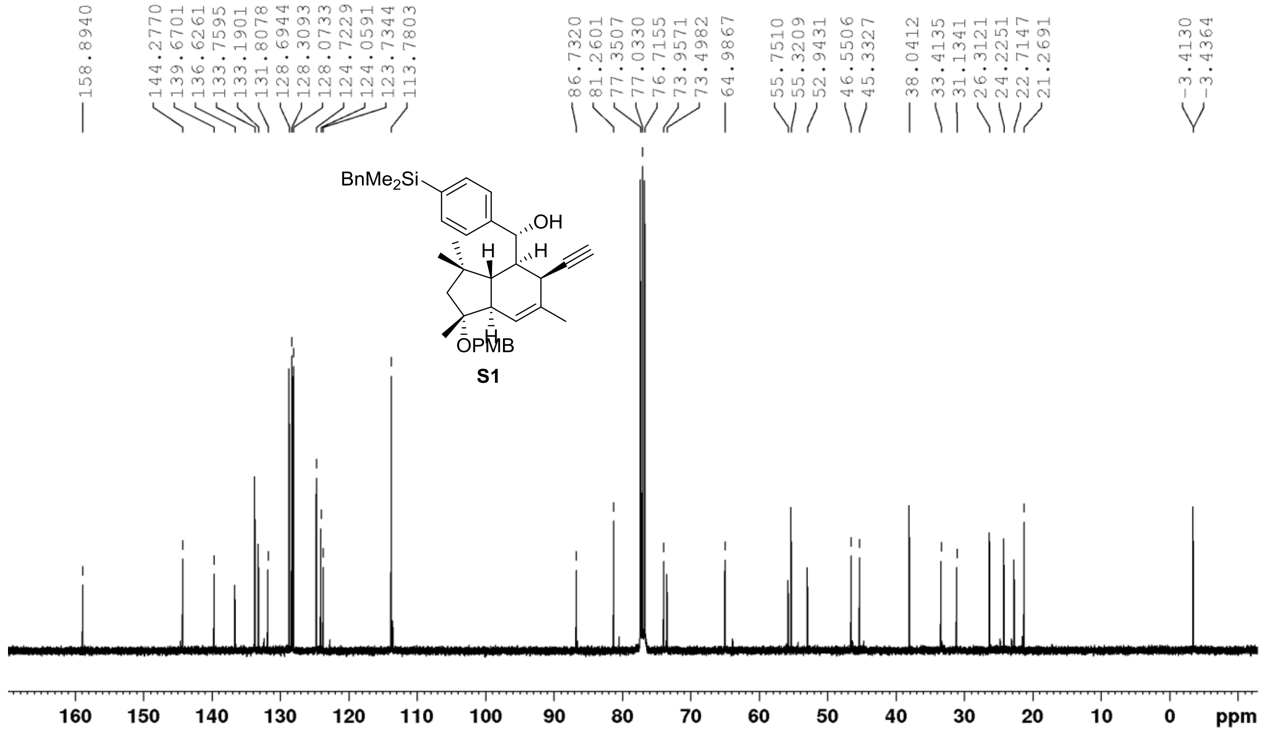
158.8940
144.2770
139.6701
136.6261
133.7595
133.1901
131.8078
128.6944
128.3093
128.0733
124.7229
124.0591
123.7344
113.7803
86.7320
81.2601
77.3507
77.0330
76.7155
73.9571
73.4982
64.9867
55.7510
55.3209
52.9431
46.5506
45.3327
38.0412
33.4135
31.1341
26.3121
24.2251
22.7147
21.2691
-3.4130
-3.4364
BnMe2Si
OH
H
H
H
OPMB
S1
160 150 140 130 120 110 100 90 80 70 60 50 40 30 20 10 0 ppm

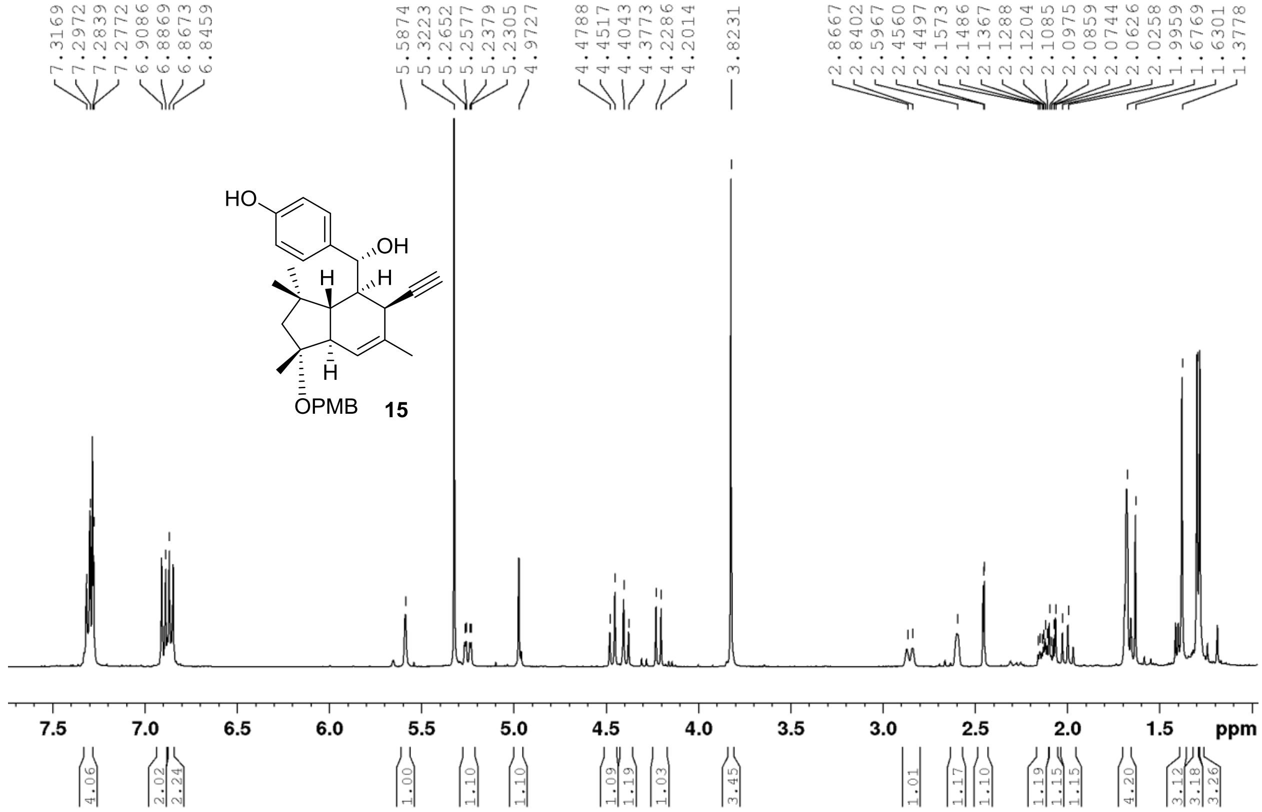
7.3169
7.2972
7.2839
7.2772
6.9086
6.8869
6.8673
6.8459
5.5874
5.3223
5.2652
5.2577
5.2379
5.2305
4.9727
4.4788
4.4517
4.4043
4.3773
4.2286
4.2014
3.8231
2.8667
2.8402
2.5967
2.4560
2.4497
2.1573
2.1486
2.1367
2.1288
2.1204
2.1085
2.0975
2.0859
2.0744
2.0626
2.0258
1.9959
1.6769
1.6301
1.3778
HO
OH
H
H
H
OPMB
15
7.5 7.0 6.5 6.0 5.5 5.0 4.5 4.0 3.5 3.0 2.5 2.0 1.5 ppm
4.06
2.02
2.24
1.00
1.10
1.10
1.09
1.19
1.03
3.45
1.01
1.17
1.10
1.19
1.15
1.15
4.20
3.12
3.18
3.26

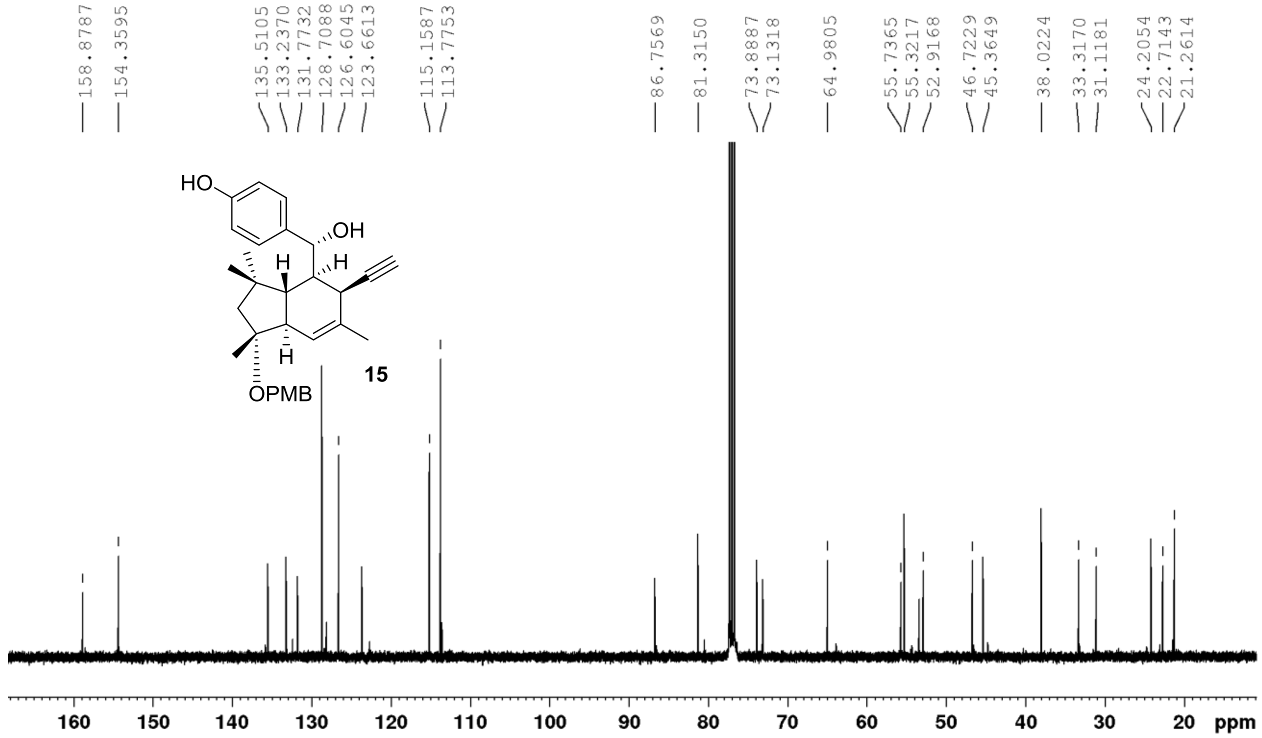
158.8787
154.3595
135.5105
133.2370
131.7732
128.7088
126.6045
123.6613
115.1587
113.7753
86.7569
81.3150
73.8887
73.1318
64.9805
55.7365
55.3217
52.9168
46.7229
45.3649
38.0224
33.3170
31.1181
24.2054
22.7143
21.2614
HO
OH
H
H
H
OPMB
15
160
150
140
130
120
110
100
90
80
70
60
50
40
30
20
ppm

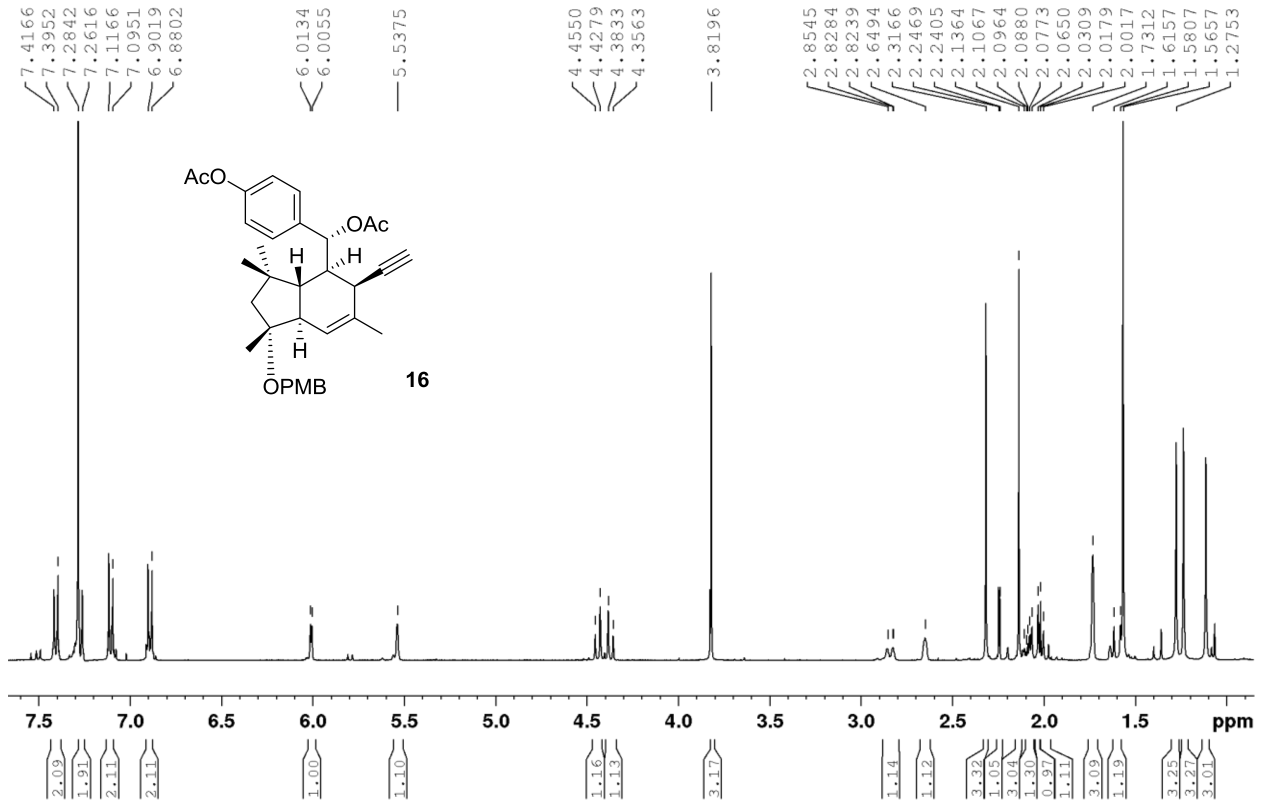
7.4166
7.3952
7.2842
7.2616
7.1166
7.0951
6.9019
6.8802
6.0134
6.0055
5.5375
4.4550
4.4279
4.3833
4.3563
3.8196
2.8545
2.8284
2.8239
2.6494
2.3166
2.2469
2.2405
2.1364
2.1067
2.0964
2.0880
2.0773
2.0650
2.0309
2.0179
2.0017
1.7312
1.6157
1.5807
1.5657
1.2753
AcO
OAc
H
H
H
OPMB
16
7.5
7.0
6.5
6.0
5.5
5.0
4.5
4.0
3.5
3.0
2.5
2.0
1.5
ppm
2.09
1.91
2.11
2.11
1.00
1.10
1.16
1.13
3.17
1.14
1.12
3.32
1.05
3.04
1.30
0.97
1.11
3.09
1.19
3.25
3.27
3.01

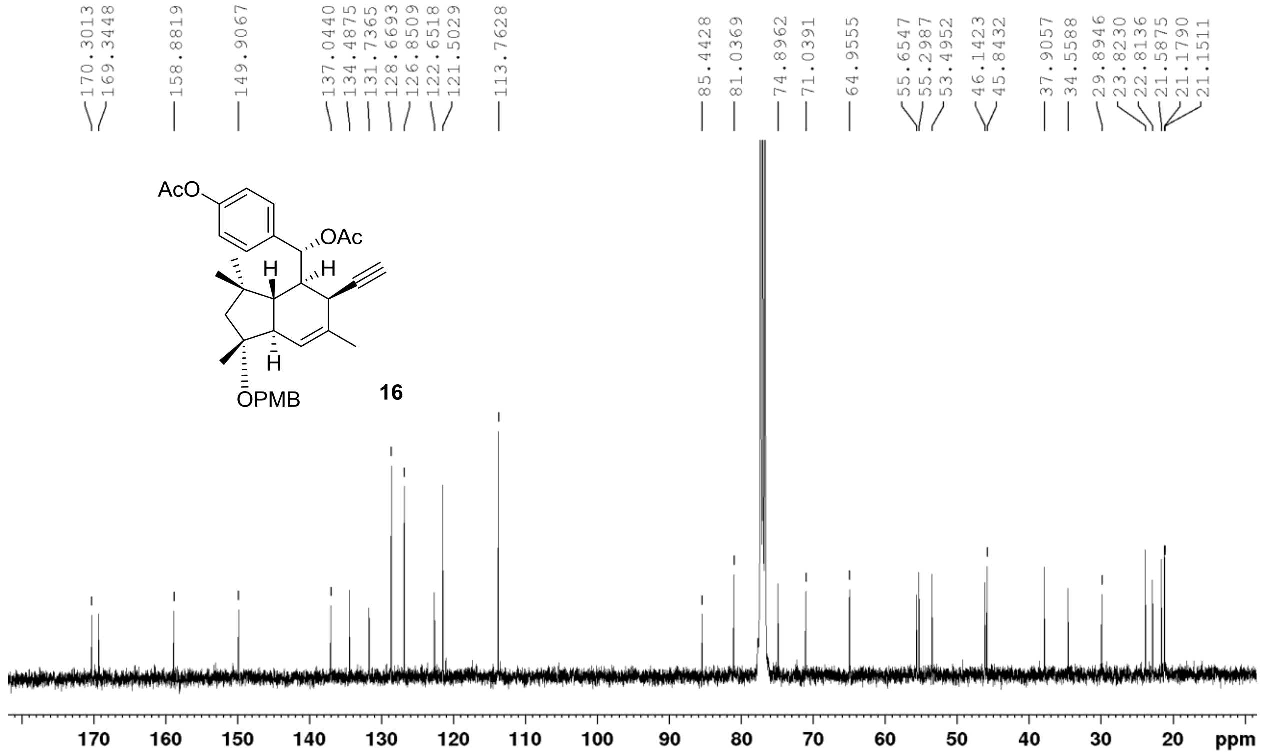
170.3013
169.3448
158.8819
149.9067
137.0440
134.4875
131.7365
128.6693
126.8509
122.6518
121.5029
113.7628
85.4428
81.0369
74.8962
71.0391
64.9555
55.6547
55.2987
53.4952
46.1423
45.8432
37.9057
34.5588
29.8946
23.8230
22.8136
21.5875
21.1790
21.1511
AcO
OAc
H
H
H
OPMB
16
170 160 150 140 130 120 110 100 90 80 70 60 50 40 30 20 ppm

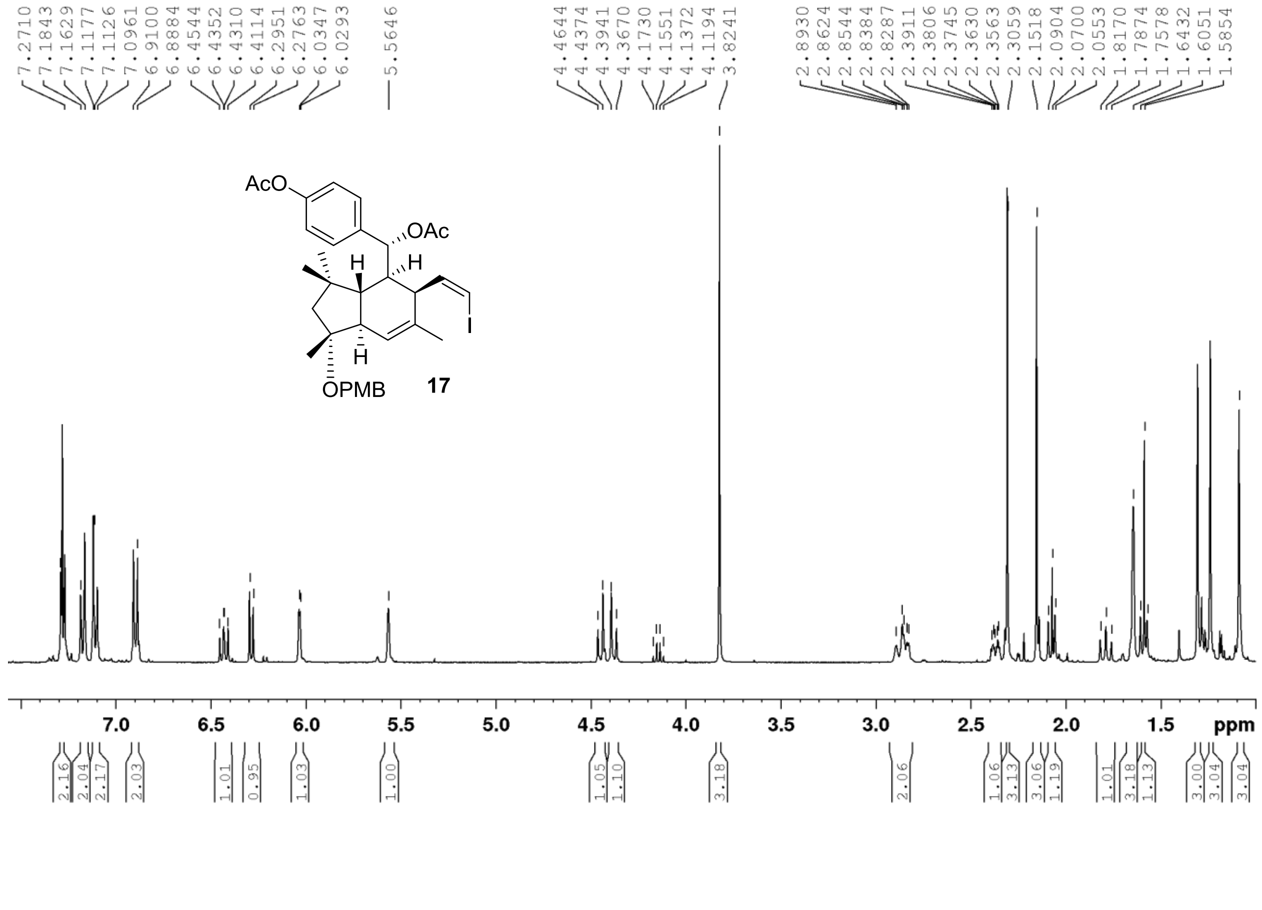
7.2710
7.1843
7.1629
7.1177
7.1126
7.0961
6.9100
6.8884
6.4544
6.4352
6.4310
6.4114
6.2951
6.2763
6.0347
6.0293
5.5646
4.4644
4.4374
4.3941
4.3670
4.1730
4.1551
4.1372
4.1194
3.8241
2.8930
2.8624
2.8544
2.8384
2.8287
2.3911
2.3806
2.3745
2.3630
2.3563
2.3059
2.1518
2.0904
2.0700
2.0553
1.8170
1.7874
1.7578
1.6432
1.6051
1.5854
AcO
OAc
H
H
H
I
OPMB
17
7.0 6.5 6.0 5.5 5.0 4.5 4.0 3.5 3.0 2.5 2.0 1.5 ppm
2.16 2.04 2.17 2.03 1.01 0.95 1.03 1.00 1.05 1.10 3.18 2.06 1.06 3.13 3.06 1.19 1.01 3.18 1.13 3.00 3.04 3.04

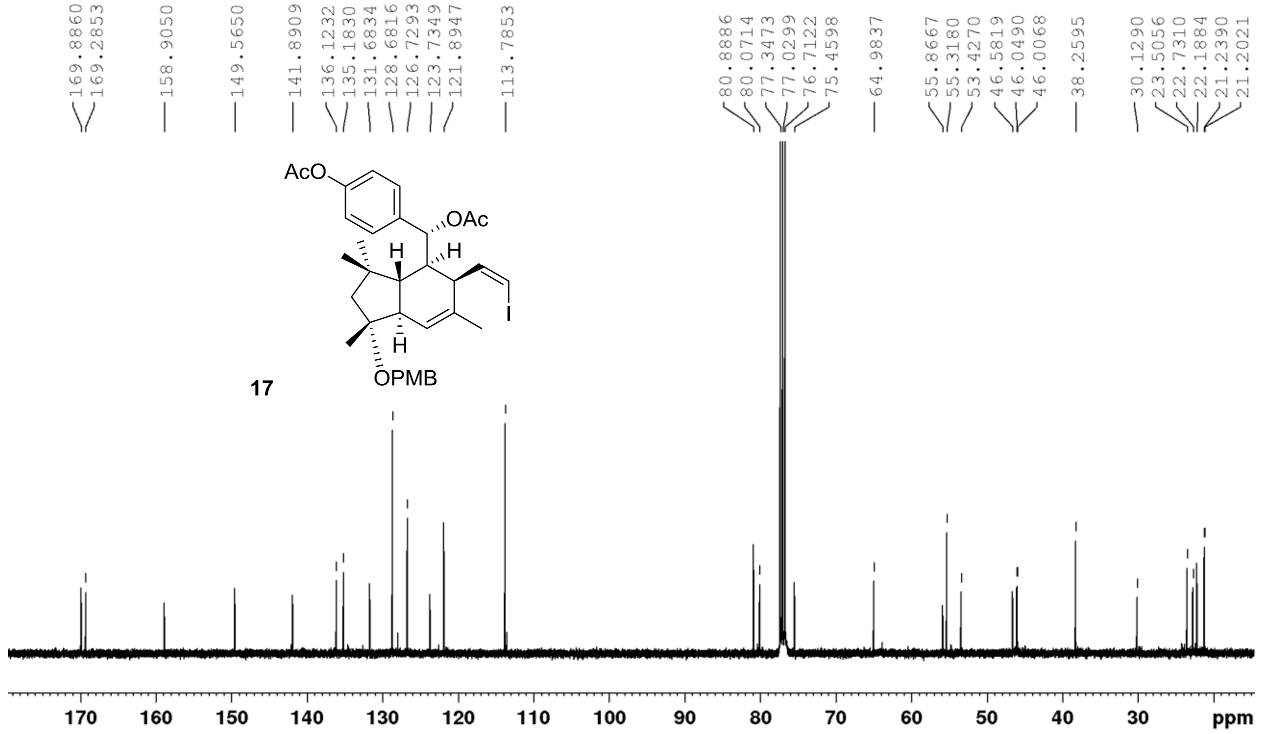
169.8860
169.2853
158.9050
149.5650
141.8909
136.1232
135.1830
131.6834
128.6816
126.7293
123.7349
121.8947
113.7853
80.8886
80.0714
77.3473
77.0299
76.7122
75.4598
64.9837
55.8667
55.3180
53.4270
46.5819
46.0490
46.0068
38.2595
30.1290
23.5056
22.7310
22.1884
21.2390
21.2021
AcO
OAc
H
H
H
I
OPMB
17
170 160 150 140 130 120 110 100 90 80 70 60 50 40 30 ppm

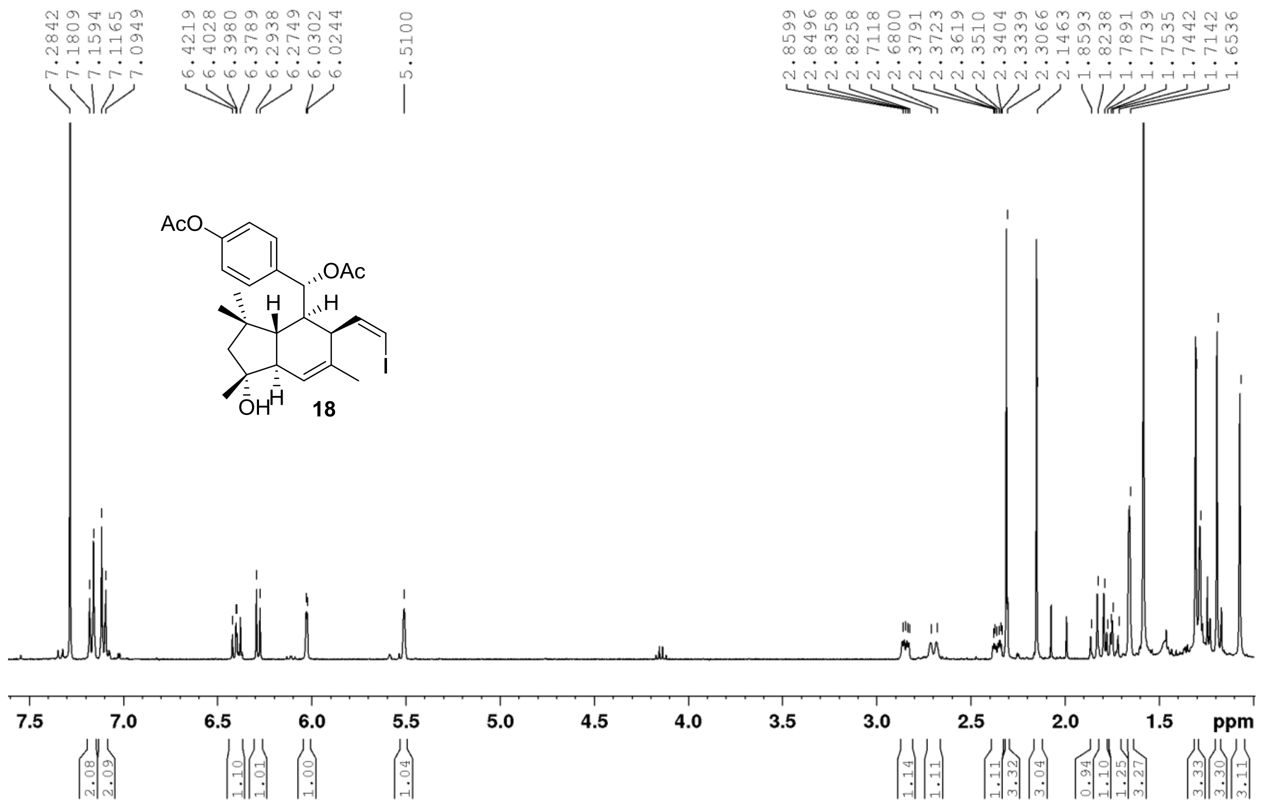
7.2842
7.1809
7.1594
7.1165
7.0949
6.4219
6.4028
6.3980
6.3789
6.2938
6.2749
6.0302
6.0244
5.5100
2.8599
2.8496
2.8358
2.8258
2.7118
2.6800
2.3791
2.3723
2.3619
2.3510
2.3404
2.3339
2.3066
2.1463
1.8593
1.8238
1.7891
1.7739
1.7535
1.7442
1.7142
1.6536
AcO
OAc
H
H
H
I
OH
18
7.5 7.0 6.5 6.0 5.5 5.0 4.5 4.0 3.5 3.0 2.5 2.0 1.5 ppm
2.08
2.09
1.10
1.01
1.00
1.04
1.14
1.11
1.11
3.32
3.04
0.94
1.10
1.25
3.27
3.33
3.30
3.11

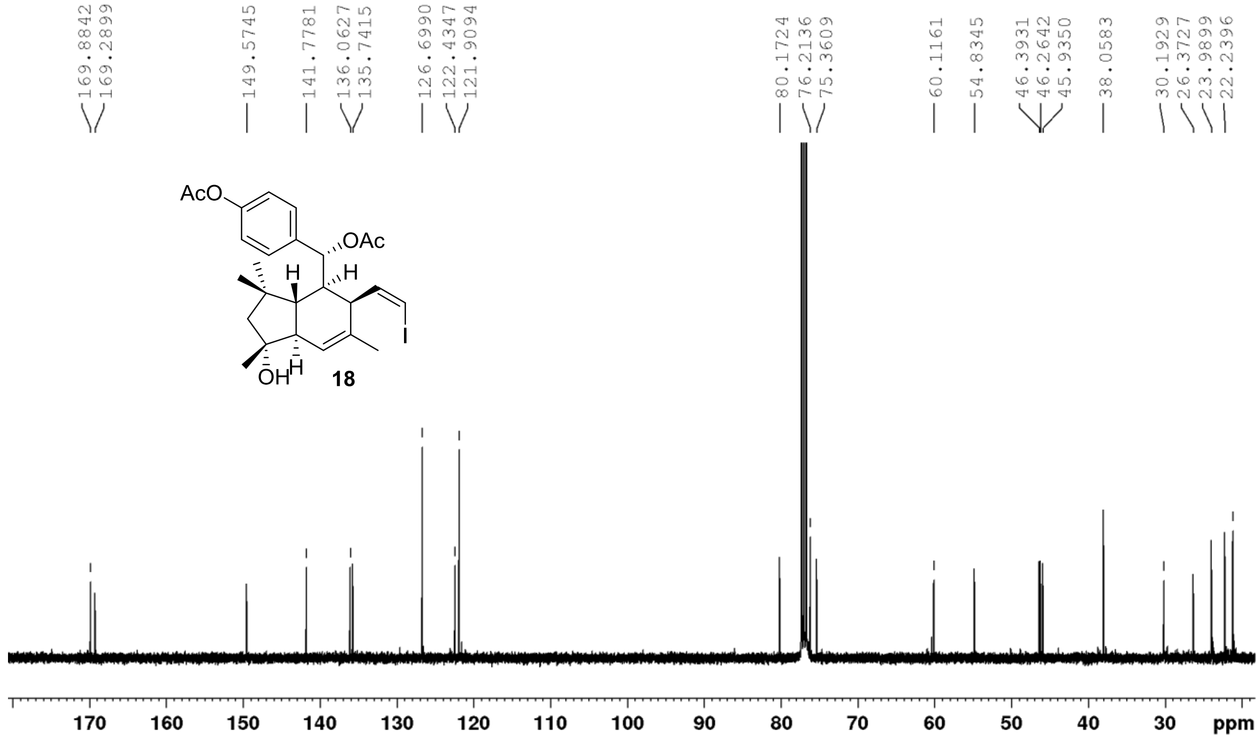
169.8842
169.2899
149.5745
141.7781
136.0627
135.7415
126.6990
122.4347
121.9094
80.1724
76.2136
75.3609
60.1161
54.8345
46.3931
46.2642
45.9350
38.0583
30.1929
26.3727
23.9899
22.2396
AcO
OAc
H
H
OH
H
I
18
170
160
150
140
130
120
110
100
90
80
70
60
50
40
30
ppm

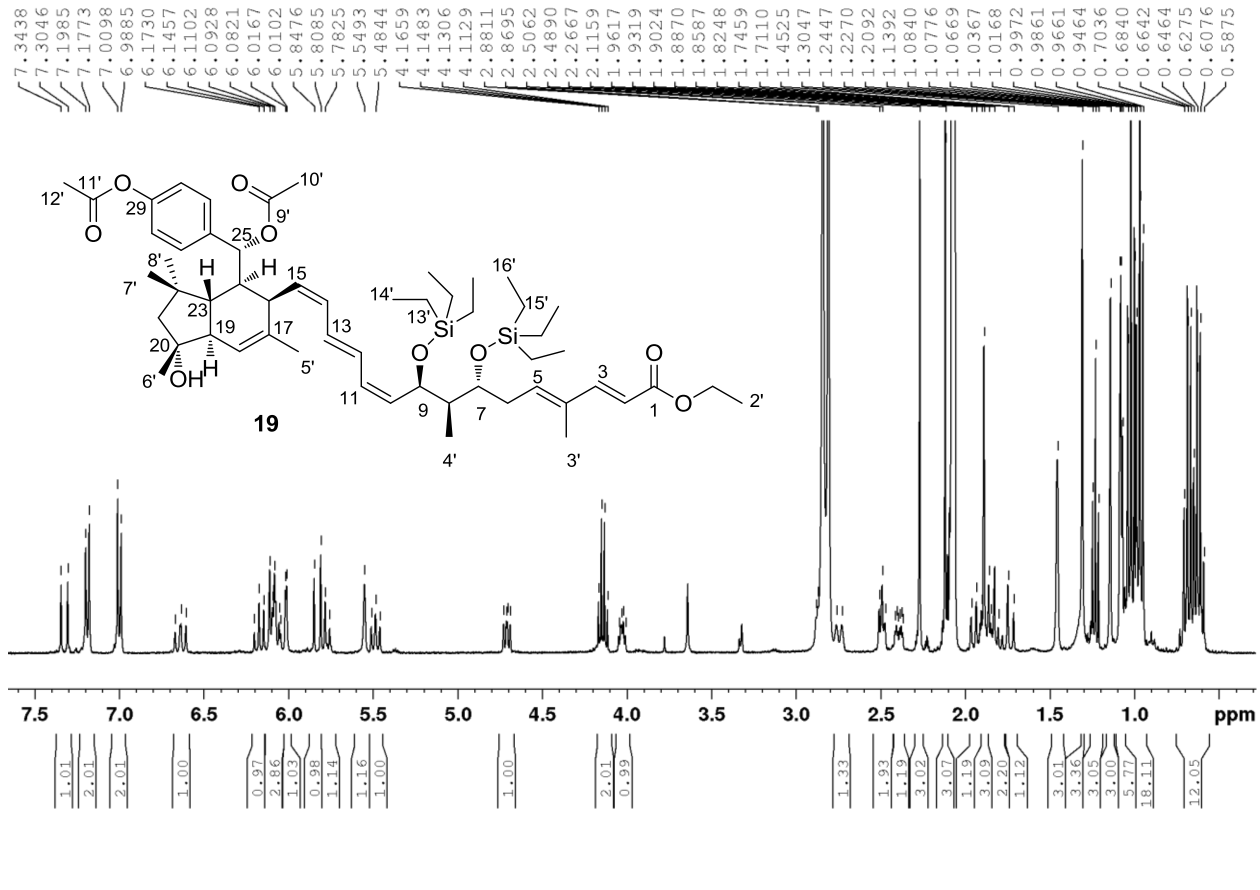
7.3438
7.3046
7.1985
7.1773
7.0098
6.9885
6.1730
6.1457
6.1102
6.0928
6.0821
6.0167
6.0102
5.8476
5.8085
5.7825
5.5493
5.4844
4.1659
4.1483
4.1306
4.1129
2.8811
2.8695
2.5062
2.4890
2.2667
2.1159
1.9617
1.9319
1.9024
1.8870
1.8587
1.8248
1.7459
1.7110
1.4525
1.3047
1.2447
1.2270
1.2092
1.1392
1.0840
1.0776
1.0669
1.0367
1.0168
0.9972
0.9861
0.9661
0.9464
0.7036
0.6840
0.6642
0.6464
0.6275
0.6076
0.5875
12'
11'
29
10'
9'
25
8'
7'
23
19
20
6'
OH
H
H
H
15
17
5'
13
11
9
7
4'
14'
13'
Si
16'
15'
Si
5
3
3'
1
2'
19
7.5
7.0
6.5
6.0
5.5
5.0
4.5
4.0
3.5
3.0
2.5
2.0
1.5
1.0
ppm
1.01
2.01
2.01
1.00
0.97
2.86
1.03
0.98
1.14
1.16
1.00
1.00
2.01
0.99
1.33
1.93
1.19
3.02
3.07
1.19
3.09
2.20
1.12
3.01
3.36
3.05
3.00
5.77
18.11
12.05

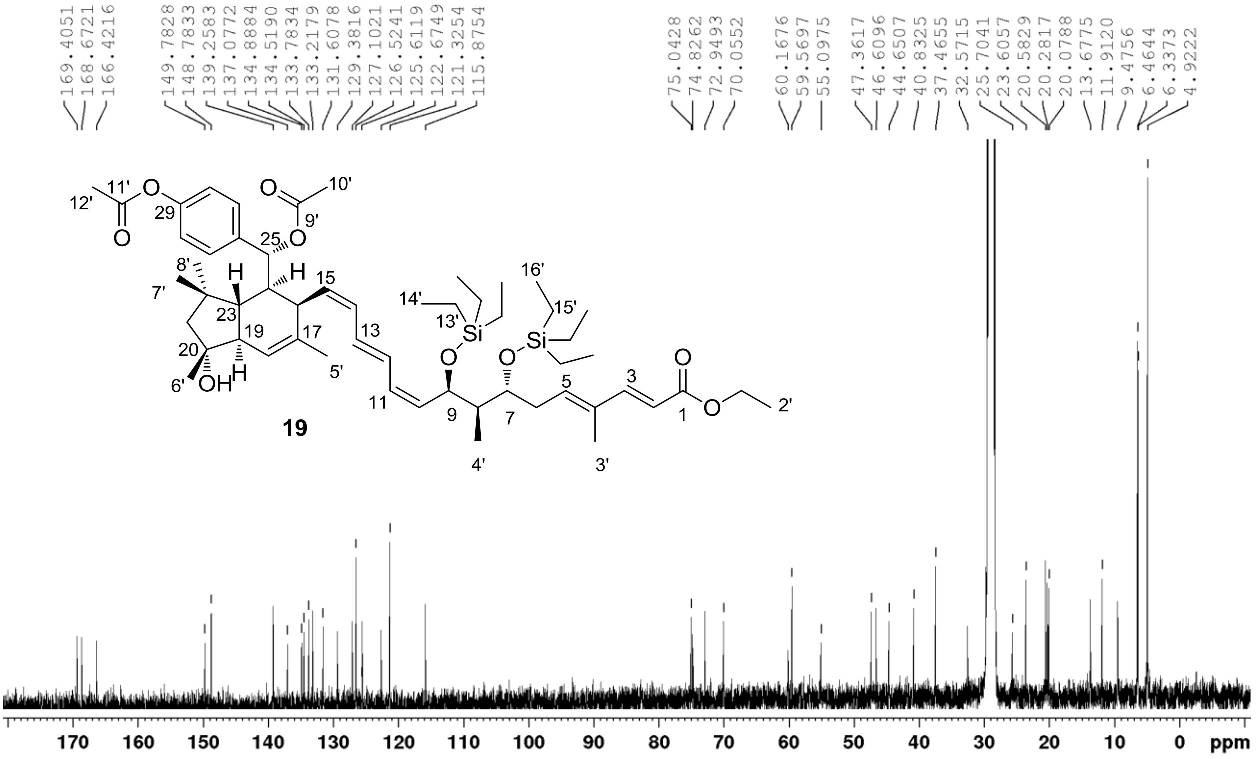

Elansolid B1: Comparison of ^{1}H NMR spectra of the synthetic sample (blue) with the reported one (red) [S1]

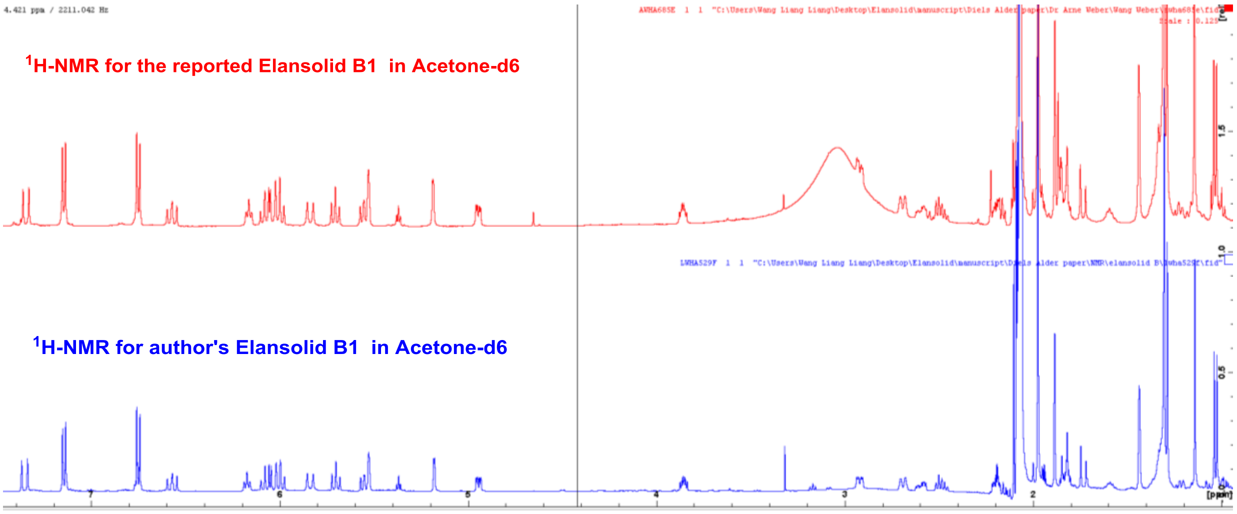

Elansolid B1: ^{1}H, ^{13}C NMR in acetone-d_6

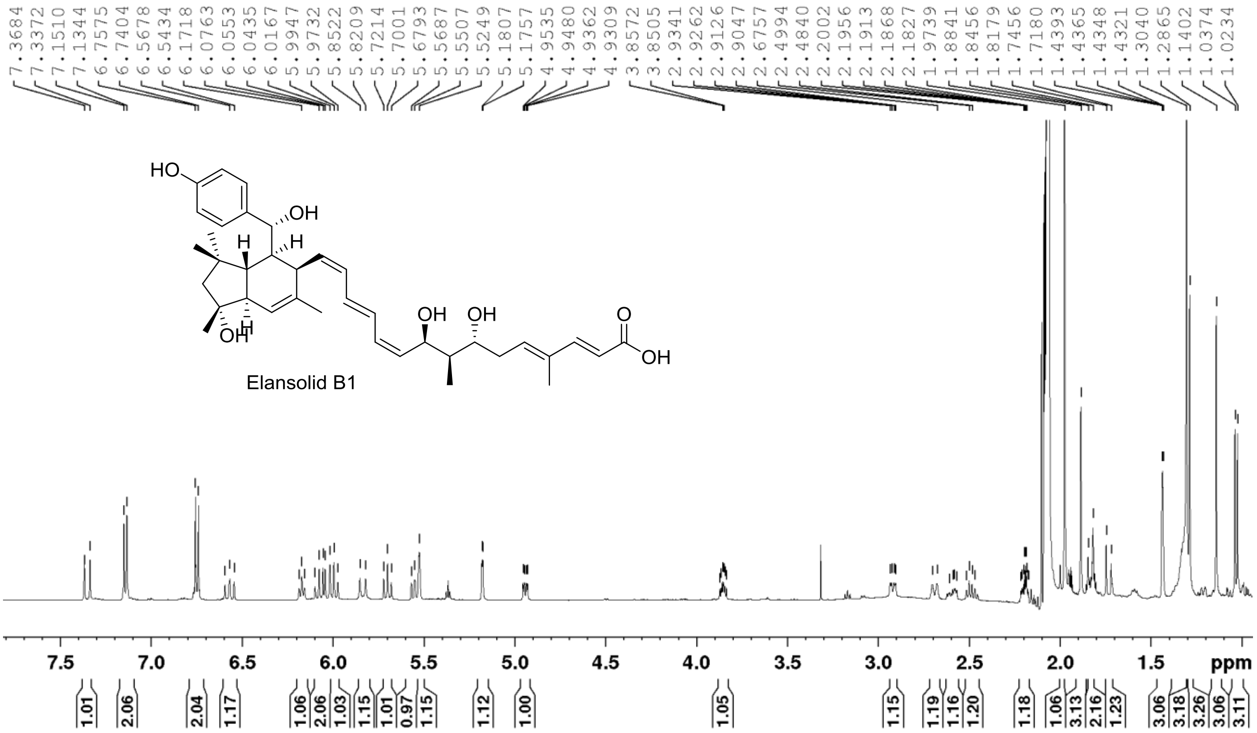

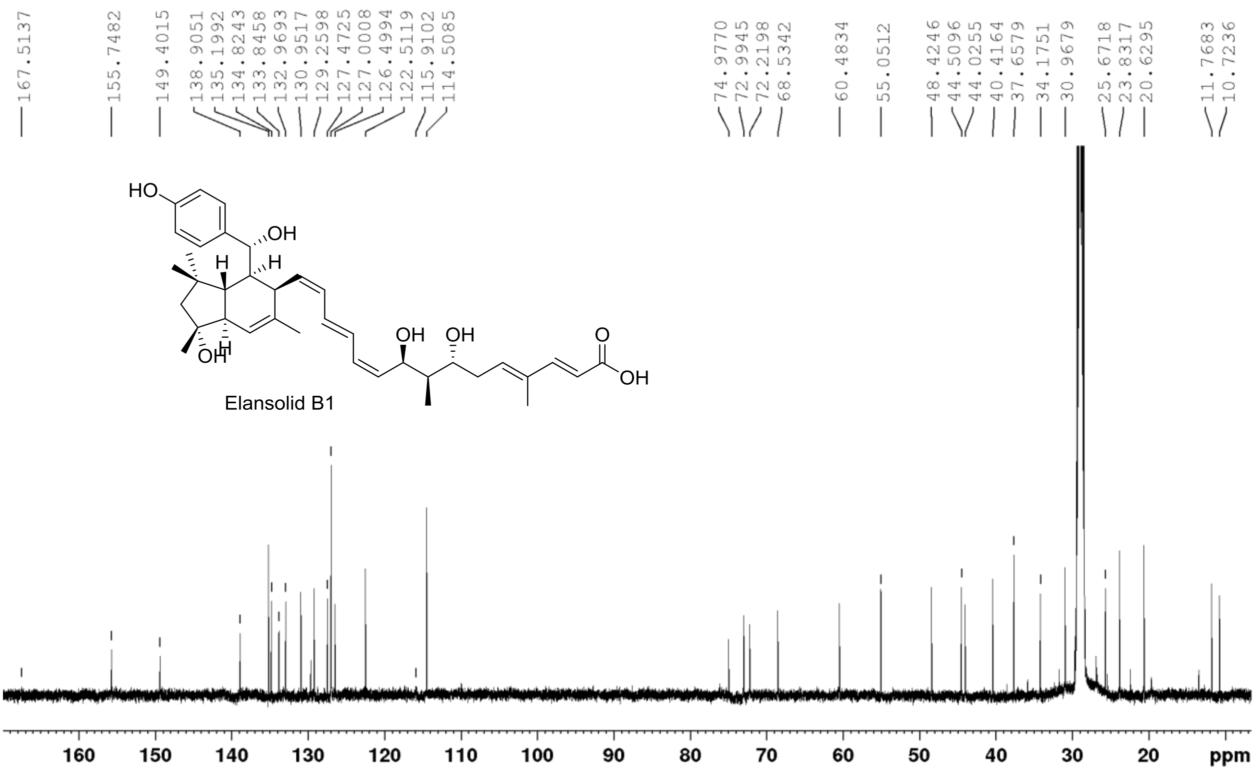

Elansolid B1:^{1}H-^{1}H COSY in acetone- d_6

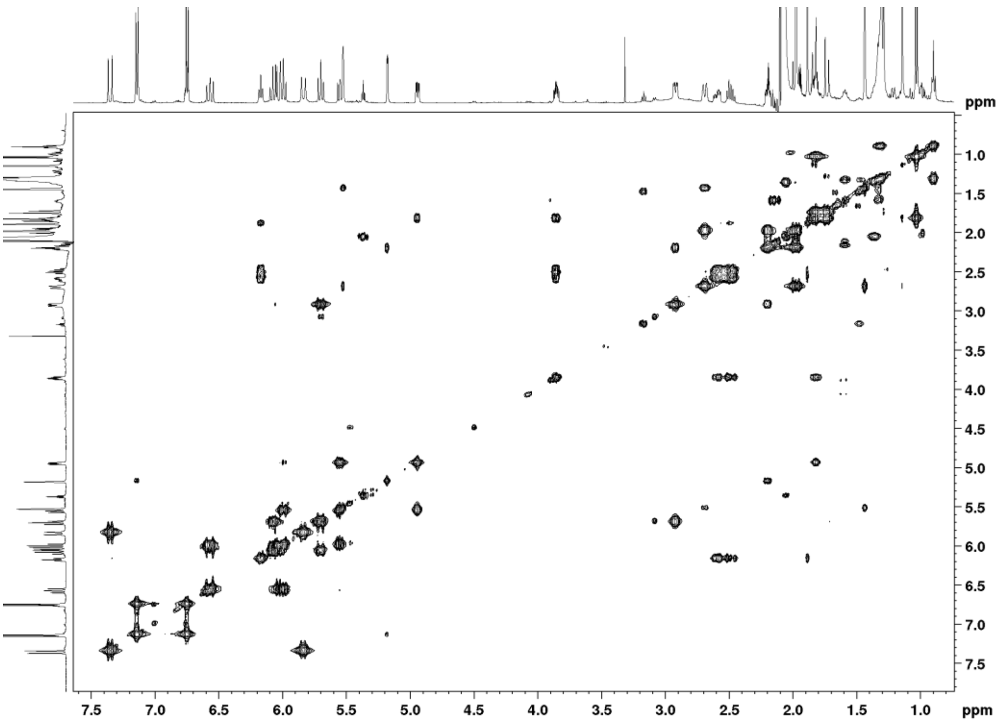

Elansolid B1: ^{1}H-^{13}C HSQC in acetone- d_6

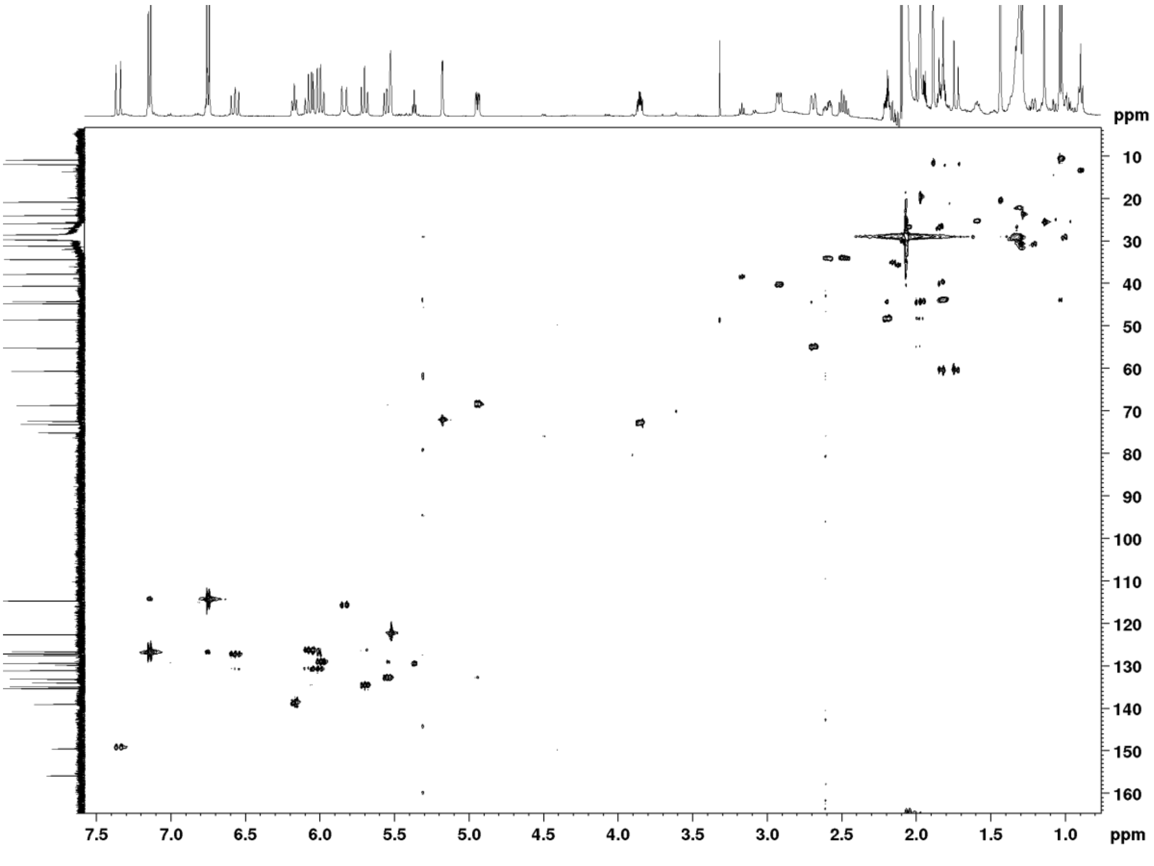

Elansolid B1: ^{1}H-^{13}C HMBC in acetone-d_6

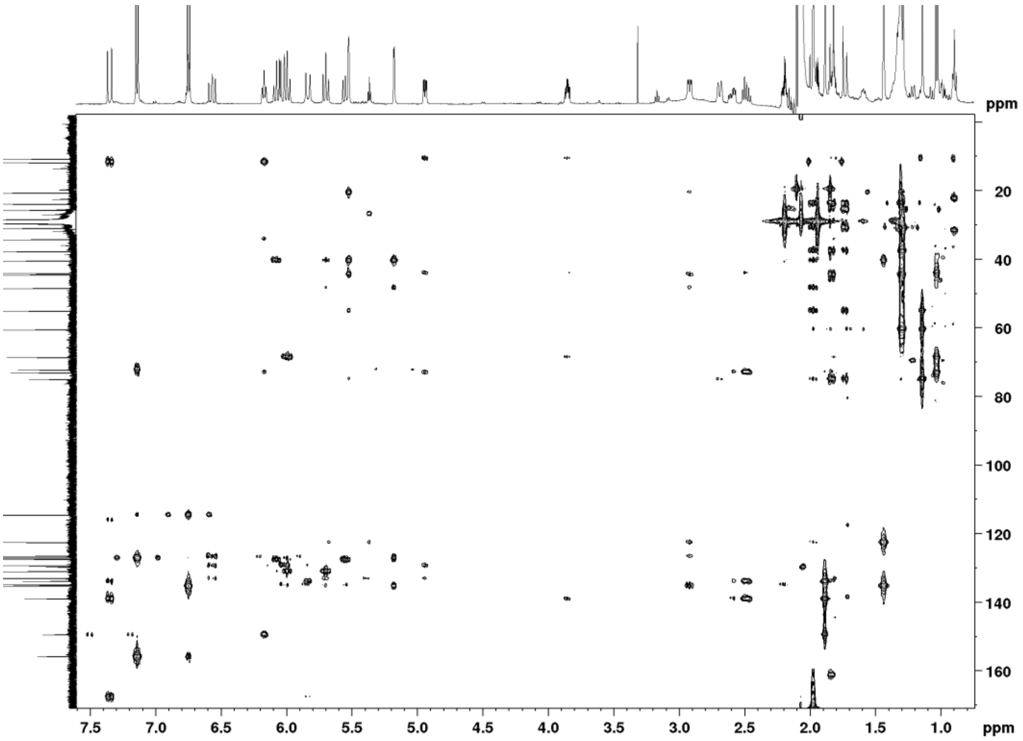

Elansolid B2: ^{1}H, ^{13}C NMR in acetone- ***d***$_{6}$

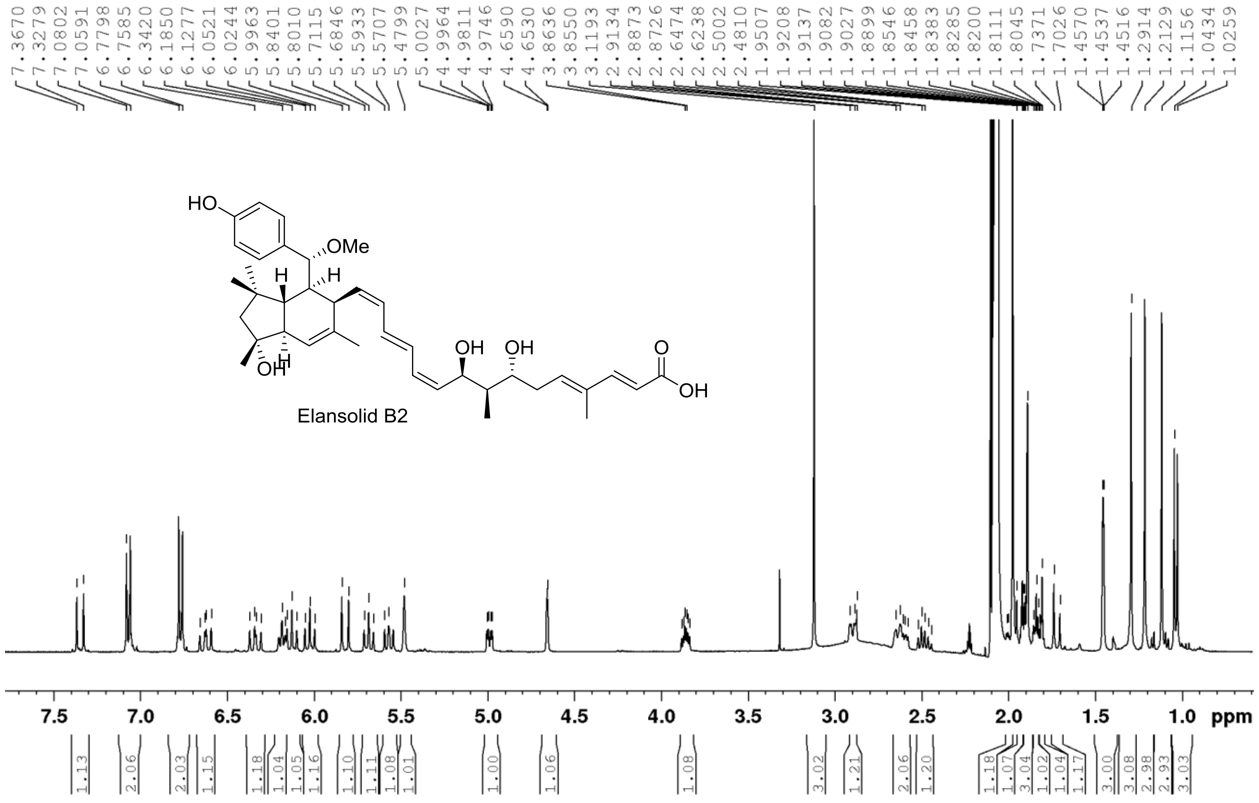

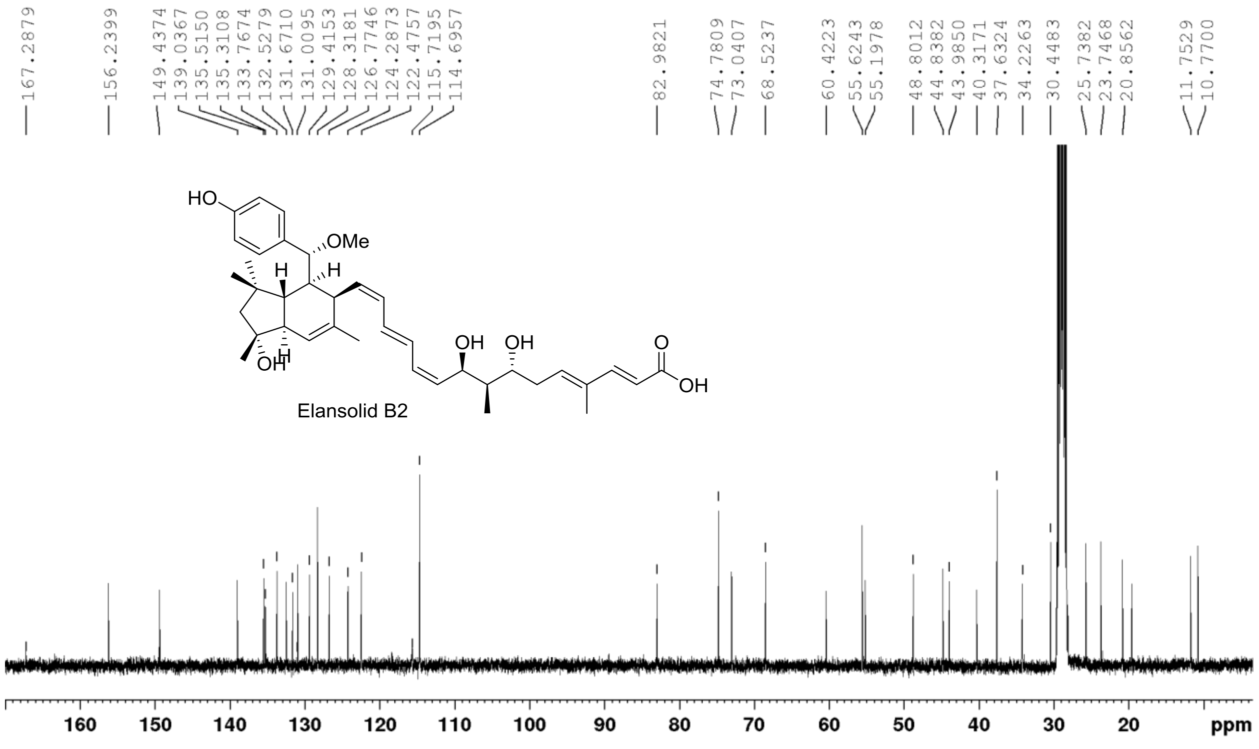

Elansolid B2:^{1}H-^{1}HCOSY in acetone- d_6

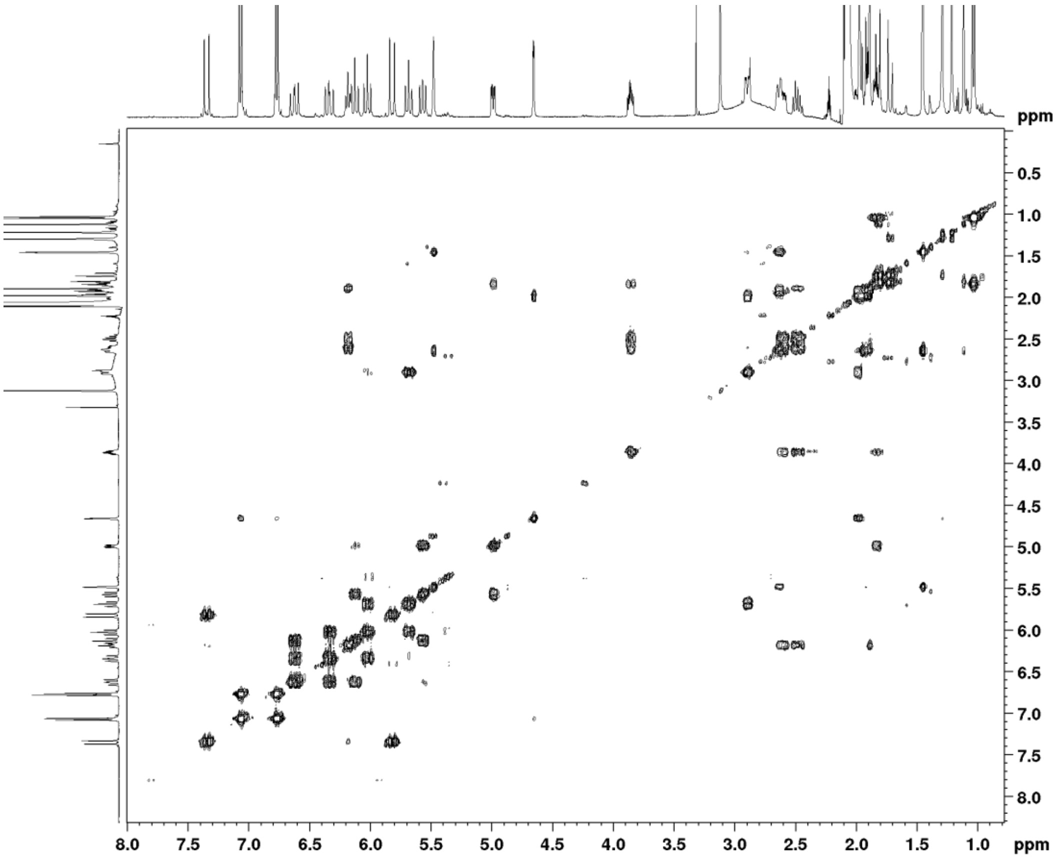

Elansolid B2: ^{1}H-^{13}C HSQC in acetone- d_6

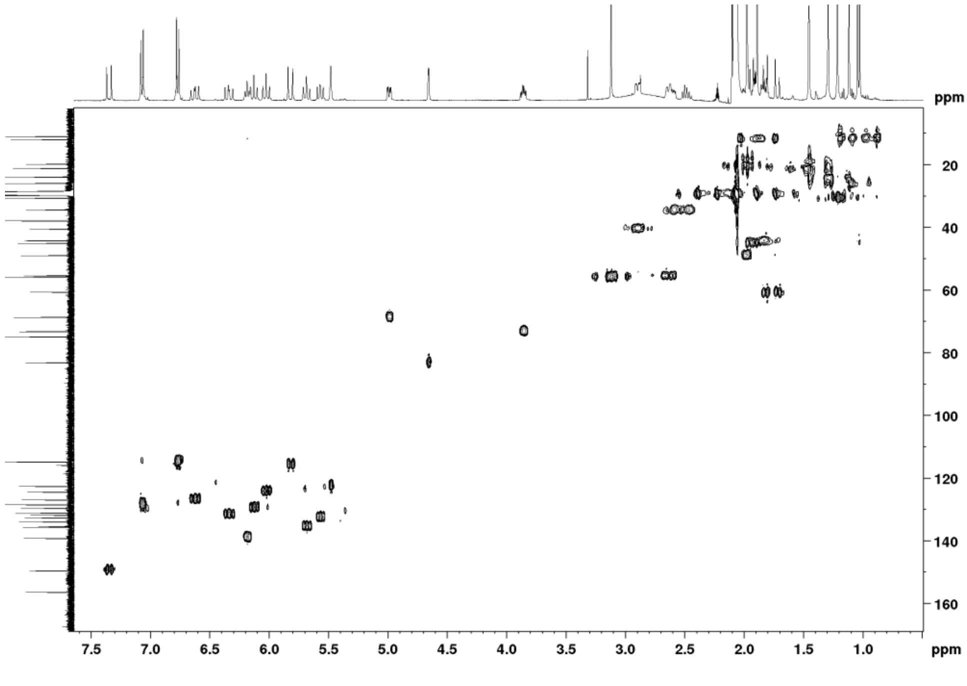

Elansolid B2: ^{1}H-^{13}C HMBC in acetone- d_6

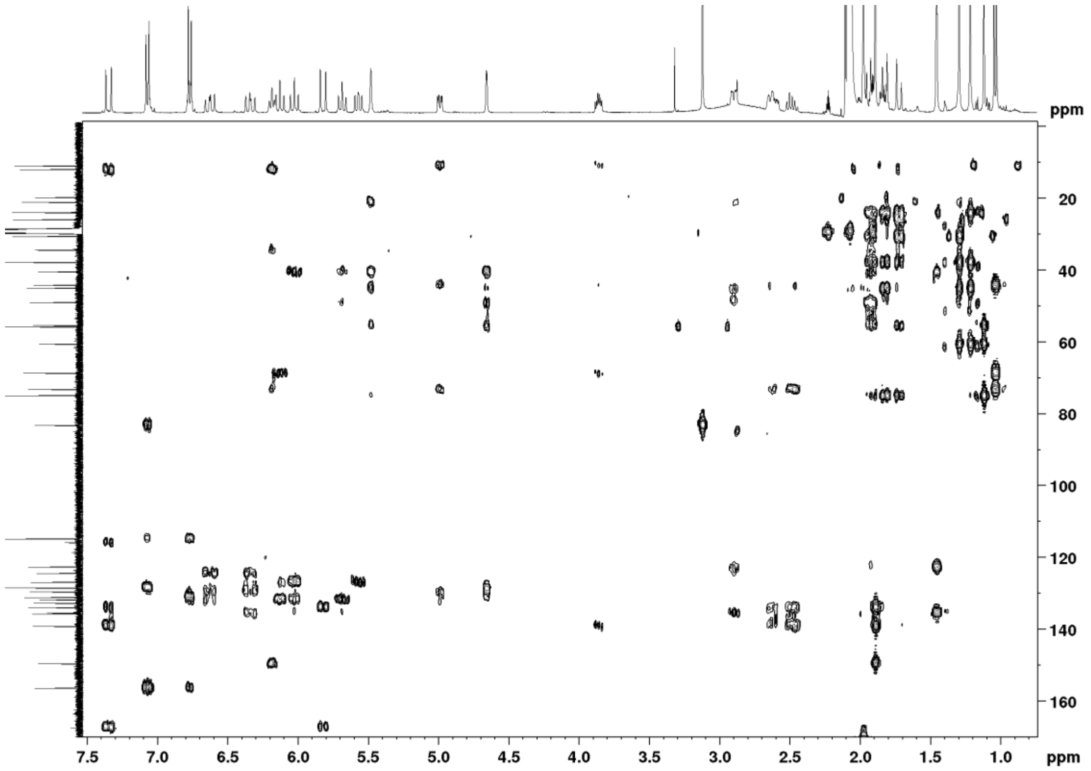

Selective key nOe for compound **19**

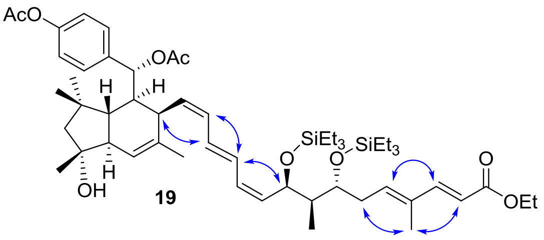

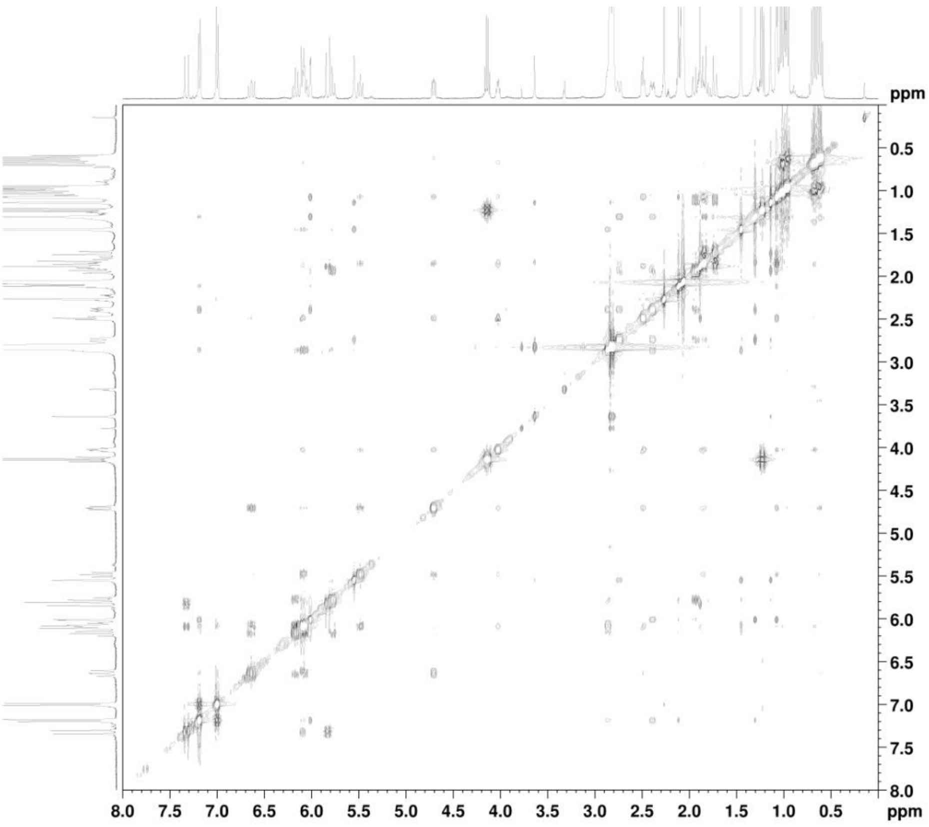

Elansolid B1: Comparison of ^{1}H, ^{13}C NMR spectra for the author's sample and the reported one

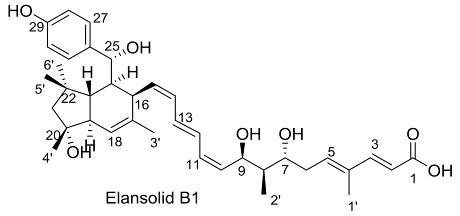

Elansolid B1

Elansolid B1: 500 MHz ^{1}H NMR (acetone-d_6) (author's sample)				Elans. B1: 600 MHZ ^{1}H NMR (acetone- d_6) (ref.: Angew. Chem. Int. Ed. 2011, 50, 532-536)			
H	δ_H	m	J(Hz)	H	δ_H	m	J(Hz)
-				-			
2	5.84	d	15.7	2	5.82	d	15.7
3	7.35	d	15.6	3	7.33	d	15.8
-				-			
5	6.17	t	7.23	5	6.17	t	7.23br
6a	2.59	ddd	4.2, 6.8, 15.3	6a	2.58	ddd	4.0,7.1, 15.3
6b	2.51-2.47	m	-	6b	2.47	dt	7.6, 15.3
7	3.87-3.84	m	-	7	3.84	td	4.2,7.2
8	1.83-1.80	m	-	8	1.80	ddq	3.7,6.6,7.0
9	4.94	ddd	0.9,3.6,8.6	9	4.93	dd	3.3,8.8br
10	5.55	dd	9.5, 10.9	10	5.53	dd	9.0, 10.5
11	5.99	dd	10.8, 11.0	11	5.98	t	11.0
12	6.57	dd	12.2, 13.6	12	6.55	dd	11.9, 14.1
13	6.09-6.02	m	-	13	6.02	t	11.7, 14.1
14	overlap			14	6.06	t	11.0
15	5.70	t	10.5	15	5.68	t	10.8
16	2.92	dd	4.0, 10.8	16	2.91	dd	4.0, 10.6
-				-			
18	5.52	s	-	18	5.51	s	br
19	2.69	d	12.6	19	2.67	dq	1.5, 12.6
-				-			
21a	1.83;	d;	13.8;	21a	1.82;	d;	13.8;
21b	1.73	d	13.8	21b	1.72	d	13.8
-				-			
23	1.99-1.95	m	-	23		dd	11.3, 12.5
24	2.22-2.18	m	-	24	2.19	ddd	3.1, 4.1, 11.5
25	5.18	d	2.5	25	5.16	d	2.6
-				-			
27	7.14	d	8.3	27	7.13	d	8.4
28	6.75	d	8.5	28	6.73	d	8.4
-				-			
1'	1.88	s	-	1'	1.87	s	-
2'	1.03	d	7.0	2'	1.01	d	7.0
3'	1.44	dd	1.4, 2.2	3'	1.42	q	1.0br
4'	1.14	s	-	4'	1.13	s	-
5'	1.30	s	-	5'	1.29	s	-
6'	1.29	s	-	6'	1.27	s	-

Elansolid B1: 125 MHz ^{13}C NMR (acetone- d_6) (author's sample)		Elans. B1: 125 MHz ^{1}H NMR (acetone- d_6) (ref.: Org.Lett. 2016, 16, 568-571)	
C	δ_C	C	δ_C
1	167.5	1	-
2	115.9	2	-
3	149.4	3	150.2
4	133.8	4	134.8
5	138.9	5	139.7
6	34.1	6	35.1
7	73.0	7	73.9
8	44.0	8	44.9
9	68.5	9	69.4
10	132.9	10	133.9
11	129.3	11	130.2
12	127.5	12	128.3
13	130.9	13	131.9
14	126.5	14	127.4
15	134.8	15	135.7
16	40.4	16	41.3
17	135.2	17	136.1
18	122.5	18	123.4
19	55.1	19	55.9
20	74.9	20	75.9
21	60.5	21	61.4
22	37.7	22	38.6
23	44.5	23	45.4
24	48.4	24	49.3
25	72.2	25	73.1
26	135.2	26	136.1
27	127.0	27	127.9
28	114.5	28	115.4
29	155.7	29	156.7
1'	11.8	1'	12.7
2'	10.7	2'	11.6
3'	20.6	3'	21.5
4'	25.7	4'	26.6
5'	30.9	5'	31.9
6'	23.8	6'	24.7

Elansolid B2: Comparison of ^{1}H, ^{13}C NMR spectra for the author's sample and the reported one

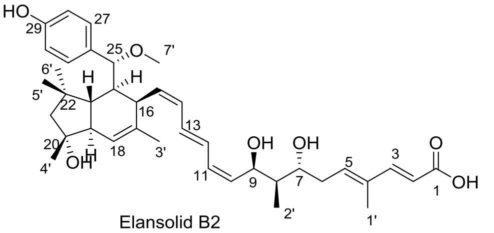

Elansolid B2: 400MHz ^{1}H NMR (acetone- d_6) (author's sample)				Elans. B2: 600MHz ^{1}H NMR (acetone- d_6) (ref.: Angew. Chem. Int. Ed. 2011, 50, 532-536)			
H	δ_H	m	J(Hz)	H	δ_H	m	J(Hz)
-				-			
2	5.82	t	15.7	2	5.80	d	15.4
3	7.35	d	15.7	3	7.33	d	15.4
-				-			
5	6.18	t	7.2	5	6.17	t	7.2br
6a	2.60-2.58	m		6a	2.59	ddd	3.7, 7, 15.4
6b	2.52-2.44	m		6b	2.47	dt	7.7, 15.4
7	3.88-3.84	m	-	7	3.85	ddd	4, 7, 8
8	1.85-1.82	m	-	8	1.81	ddq	3.7, 6.8, 7.2
9	4.99	ddd	0.9, 3.5, 8.6	9	4.98	ddd	1, 3.2, 8.8
10	5.57	dd	9.7, 10.1	10	5.55	dd	8.8, 11
11	6.13	t	11.2	11	6.11	t	11.2
12	6.63	dd	11.5, 14.6	12	6.61	dd	11.5, 14.6
13	6.34	dd	11.5, 14.6	13	6.32	dd	11.5, 14.6
14	6.02	t	11.2	14	6.01	t	11.2
15	5.68	t	10.8	15	5.67	t	10.8
16	2.90	dd	3.3, 10.6	16	2.88	dd	3.5, 10.5
-				-			
18	5.48	s	-	18	5.47	s	-
19	2.64-2.62	m	-	19	2.63	dtq	1.8, 2, 12.1
-				-			
21a	1.82	d	13.5	21a	1.81	d	14.0
21b	1.72	d	13.8	21b	1.71	d	14.0
-				-			
23	1.95-1.91	m	-	23	1.91	t	12.2
24	2.01-1.97	m	-	24	1.97	m	2.6, 3.8, 11.4
25	4.66	d	2.4	25	4.64	d	2.6
-				-			
27	7.07	d	8.4	27	7.05	d	8.5
28	6.77	d	8.5	28	6.75	d	8.5
-				-			
1'	1.89	s	-	1'	1.87	s	-
2'	1.03	d	7.0	2'	1.02	d	7.0
3'	1.45	dd	1.3, 2.1	3'	1.44	dt	1, 1.3
4'	1.12	s	-	4'	1.10	s	-
5'	1.29	s	-	5'	1.28	s	-
6'	1.21	s	-	6'	1.20	s	-
7'	3.12	s	-	7'	3.10	s	-

Elansolid B2: 100 MHz ^{13}C NMR (acetone- d_6) (author's sample)		Elansolid B2: 150 MHz ^{13}C NMR (acetone- d_6) (ref.:Angew. Chem. Int. Ed. 2011, 50, 532-536)	
C	δ_C	C	δ_C
1	167.3	1	168.1
2	115.7	2	116.5
3	149.4	3	150.4
4	133.8	4	134.7
5	139.0	5	139.9
6	34.2	6	35.2
7	73.0	7	74.1
8	44.0	8	44.9
9	68.5	9	69.5
10	132.5	10	133.4
11	129.4	11	130.3
12	126.8	12	127.7
13	131.6	13	132.6
14	124.3	14	125.2
15	135.5	15	136.4
16	40.3	16	41.2
17	135.3	17	136.2
18	122.5	18	123.3
19	55.2	19	56.1
20	74.8	20	75.8
21	60.4	21	61.4
22	37.6	22	38.5
23	44.8	23	45.8
24	48.8	24	49.7
25	82.9	25	83.9
26	131.0	26	131.9
27	128.3	27	129.2
28	114.7	28	115.6
29	156.2	29	157.2
1'	11.8	1'	12.6
2'	10.8	2'	11.6
3'	20.9	3'	21.7
4'	25.7	4'	26.6
5'	23.7	5'	24.6
6'	30.4	6'	31.3
7'	55.6	7'	56.5

4. References SI:

[S1] A. Weber, R. Dehn, N. Schläger, B. Dieter, A. Kirschning, *Org. Lett.* **2014**, *16*, 568-571.

[S2] L.L.Wang, D. Candito, G. Dräger, J. Hermann, R. Müller, A. Kirschning, *Chem. Eur. J.* **2017**, DOI:10.1002/chem.201605884.
